# Acute murine cytomegalovirus infection boosts cell-type specific response and lipid metabolism changes in the liver of infant mice

**DOI:** 10.3389/fimmu.2023.1169869

**Published:** 2023-08-10

**Authors:** Juanzi Gao, Anmin Wang, Xiangyi Bu, Weidong Jia

**Affiliations:** ^1^ Department of Hepatic Surgery, The First Affiliated Hospital of University of Science and Technology of China (USTC), Division of Life Sciences and Medicine, University of Science and Technology of China, Hefei, China; ^2^ Institute of Immunology, the Chinese Academy of Sciences Key Laboratory of Innate Immunity and Chronic Disease, School of Basic Medical Sciences, Division of Life Sciences and Medicine, University of Science and Technology of China, Hefei, China; ^3^ Anhui Province Key Laboratory of Hepatopancreatobiliary Surgery, The First Affiliated Hospital of University of Science and Technology of China (USTC), Hefei, China

**Keywords:** MCMV: murine CMV, liver, single cell sequencing, infant mice, immune response

## Abstract

**Introduction:**

Human cytomegalovirus (HCMV) infection in infants can lead to severe diseases, including neonatal hepatitis. The single-cell dimensional changes in immune cells after the initial CMV infection remain elusive, as do the effects of CMV infection on hepatic lipid metabolism.

**Methods:**

We employed single-cell RNA-sequencing to investigate the changes in liver cell types and immune responses in infant mice following murine CMV (MCMV) infection. Additionally, we examined alterations in protein expression profiles related to lipid metabolism in hepatocytes and the role of the key transcription factor PPAR-γ in hepatocytes during CMV infection.

**Results:**

Our study revealed that MCMV infects most liver cell types in infant mice, leading to an increase in the proportion of proliferating CD8 effector T cells and a subset of Nos2^+^ monocytes, potentially playing an essential role in early anti-viral responses. Furthermore, MCMV infection resulted in altered protein expression of lipid metabolism in hepatocytes. Knocking down the transcription factor PPAR-γ in hepatocytes effectively inhibited CMV infection.

**Discussion:**

Our findings underscore the immune system's response to early-stage MCMV infection and the subsequent impact on hepatic lipid metabolism in infant mice. This research provides new insights into the mechanisms of CMV infection and could pave the way for novel therapeutic strategies.

## Introduction

Human cytomegalovirus (HCMV) is an enveloped, double-stranded DNA virus of the family herpesviridae, subfamily β-herpesvirus, and genus cytomegalovirus (1). Seroprevalence is high across human populations, ranging from 50% to 90% (2, 3). Acquired CMV infection is asymptomatic in a majority of immunocompetent hosts; however, its effects in infants and immunocompromised patients are severe (4). Due to an immature and developing immune system, newborns have an increased vulnerability to infectious disease, including hepatitis, a common complication of CMV infection, especially neonatal hepatitis (5).

The innate immune system consists of granulocytes (mainly neutrophils), antigen-presenting cells (APCs), natural killer (NK) cells, and γδ-T cells, which are highly effective against a wide range of pathogens (6, 7). Due to limited antigen exposure *in utero*, neonates rely on innate immune responses to protect themselves from infection (6). However, previous studies have demonstrated that human infants can mount adult-like protective adaptive immune responses to viral infections and vaccines (8). Although the heterogeneity and antiviral effectiveness of CMV-specific immune response in adults are well known, the single-cell dimensional mechanism of immune cell alteration in infant mice with the first CMV infection is still unknown (9).

Hepatocytes play an important role in lipid metabolism. When CMV is not effectively controlled by a healthy immune response, replication of CMV occurs in hepatocytes and bile ducts, causing liver injury and interruption of lipid metabolic enzymatic expression and activity (10). Although studies have confirmed the correlation between CMV infection and a variety of diseases including atherosclerosis, metabolic syndrome, diabetes, and liver cholestasis, the impact of CMV infection on neonatal hepatocytes is still unclear (11–15).

Murine CMV (MCMV) infection resembles HCMV (16–18), which is a useful tool for exploring early immune response in infants after contracting CMV infection. Based on single-cell RNA-sequencing, we found that MCMV can infect most liver cell types in infant mice, especially epithelial cells, and increase the percentage of proliferating CD8 effector T cells and a subset of Nos2^+^ monocytes; these cells may be vital in the early anti-viral process. Further analysis characterized the overall changes in protein expression profile during lipid metabolism in hepatocytes after MCMV infection.

## Results

### MCMV infection increased the percentage of Kupffer cells and monocytes and decreased the percentage of T cells in the liver of infant mice

**Figure 1:**
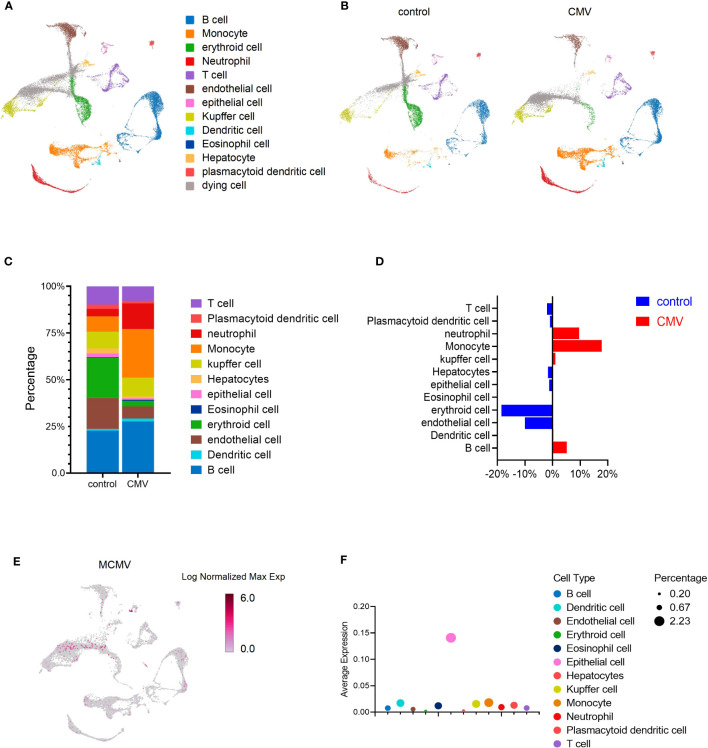
MCMV infection changes the profile of immune cells in infant mice liver. **(A)** UMAP of 33,311 liver cells from uninfected and infected infant mice (2 weeks). Mice were infected intra-peritonelly by MCMV for 3 days. The livers from three uninfected and three infected C57BL/6J mice were combined. **(B)** UMAP of 16,685 cells from uninfected mice liver versus 16,626 cells from infected mice liver. **(C, D)** The quantified percentage of each cluster from uninfected and infected mice. **(E)** Distribution of cells containing MCMV genes in 16,626 cells from infected mice liver. **(F)** Average expression of MCMV genome in main clusters from **(A)**.

To evaluate responses to viral infection in the liver of infant mice, we infected 2-week-old C57BL/6J mice with MCMV Smith strain intraperitoneally and isolated the total liver cells from either infected mice or uninfected littermate control. The transcriptome of individual liver cells identified 13 distinct cell clusters (**Figure 1A**). Expression signatures were defined for each cell type, and representative genes were used to identify each cluster (**Figures 1A**, **S1A**). By incorporating the AddModuleScore approach, we were able to assess the cell types’ response to the signature pattern, providing an additional level of confidence in our cell type identification (**Figure S1B**). To determine whether MCMV infection induced any global changes in cell clusters based on transcriptional responses, liver cell clusters from infected mice were compared with those from uninfected mice (**Figures 1B–E**). The viral infection did not result in complete loss or gain of any specific cell type (**Figures 1B–E**). While analyzing the subtle changes in the immune cell, we observed that, overall, a relative increase in the Monocytes was the highest (**Figures 1B–D**), Neutrophils and B cells were also increased in percentage; this was further confirmed by flow cytometry (**Figure S2A**) and statistically validated by scDC (**Figure S2B**). With further analysis of the MCMV genome expression in the infected sample, we found that MCMV can infect all kinds of cell types in infant mice liver, especially epithelial cells (**Figures 1E, F**).

### MCMV infection increased the percentage of proliferating CD8 effector T cells in the liver of infant mice

T cells play an important role in the body’s response to CMV infection (9). During viral infection, naive CD8 effector T cells expand and differentiate into heterogeneous subpopulations, which have different functions including antigenic specificity, memory potential, and effector function (9, 19). Subsequently, the majority of T cells gradually decrease with a decline in antigen levels in the body and form pools with long-term memory capacity to overcome pathogen re-infection (9).

**Figure 2:**
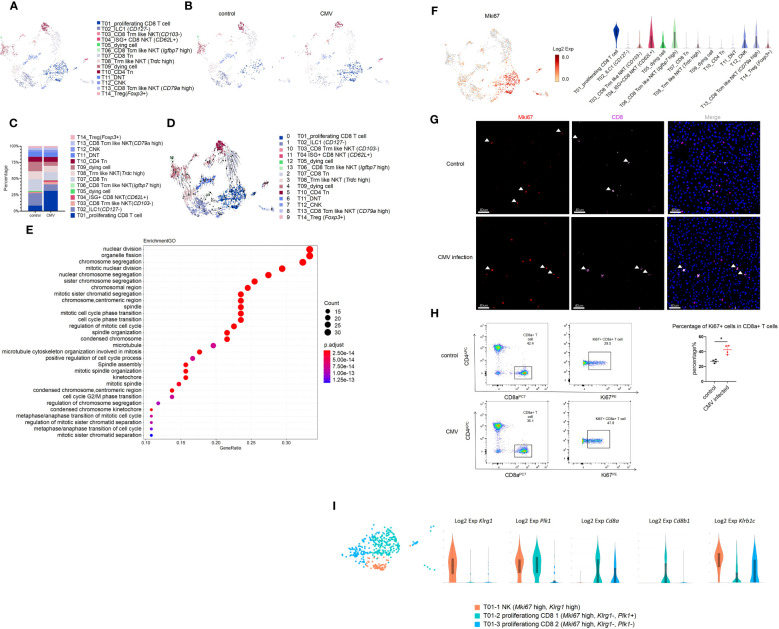
MCMV infection increases proliferating CD8 effector T cells in infant mice liver. **(A)** The T cell cluster in **Figure 1A** were re-clustered. UMAP of combined sub-clustered T cells. **(B)** UMAPs of 1,209 T cells from uninfected mice versus 906 cells from infected mice. **(C)** The quantified percentage of each cluster of combined sub-clustered T cells between uninfected and infected mice. **(D)** RNA velocities in scRNA-seq data of combined T cells subclusters derived from control and infected mice liver. **(E)** Enriched GO terms for significantly up-regulated genes in cluster T01_proliferating CD8 T cell compared with other sub-clusters in T cell cluster of MCMV infected mice liver. **(F)** Distribution and expression of proliferating marker *Mki67*. **(G)** Experimental infant mice (2 weeks) were infected with MCMV by intraperitoneal injection of 5×10^6^ TCID50 in 100ul DMEM for 3 days. Livers were isolated from wild-type C57BL/6J mice infected by MCMV, as well as the wild-type littermate control. Immunofluorescent labeling of CD8+ T cells(pink) and MKI67+ proliferating cells (red) were shown. Cells nuclei were visualized with 4,6-diamidino-2-phenylindole (DAPI, Invitrogen). White arrowheads indicate double-labeled cells (Scale bars, 40 µm). Experiments in **(G)** were repeated twice. **(H)** Representative flow cytometry (FC) plots and quantification showing the percentage of Ki67+ cells in CD8+ T cells in the liver from indicated mice (n = 4, mean ± SD). Statistical significance was determined by non-parametric Mann Whitney test between groups (*P < 0.05). Experiments were repeated two times. **(I)** Expression level of marker genes of sub-clusters within T01 proliferating *Mki67*+ T cell.

We focused on the acute phase of the infection process. Although the total percentage of T cells remained constant, we explored the changes in T cell sub-clusters. Based on known gene signatures, T cells were segregated into 14 clusters by unsupervised clustering (**Figures 2A, B**). In CD3+ cells, we identified T10 and T14 as CD4+ T cells, T01 and T07 as CD8+T cells (*Cd8a*+, *Cd8b1*+), and T11 as CD4-CD8- cell. In addition, we identified T03, T04, T06, T08, and T13 as CD8αα+ Natural Killer T (NKT) cells for expression of NK1.1 (*Klrb1c*) and *Cd8a* in them (**Figure S3A**). In CD3- cells, we defined T02 as a type 1 innate lymphoid cells (ILC1) cell and T12 as a conventional natural killer (cNK) cell based on the expression of *Cd49a* and *Cd49b* (**Figure S3A**). It was worth noting that only *Cd127*- *Tcf7*- “cytotoxic-like” effector ILC1 cells, but not *Cd127*+ “helper-” early maturation ILC1 cells were detected in the liver of infant mice (**Figure S3B**) (20). Moreover, unlike adult mice, we found that ILC1 was significantly more abundant than cNK in infant mice, as previously reported (**Figures 2C**, **S3C**) (21).

For CD4 T cell (*Cd4*+)(T10, T14), based on the expression of *Cd44* and *Cd62l* (*Sell*), we defined T10 CD4 T cells as naive T cells (Tn, *Cd44*-, *Cd62l*+) (**Figures S3A**, **D**) (22). Based on the expression of *Foxp3*, we defined T14 CD4 T cells as *Cd44*+ Treg cells, and we found that the percentage of these two groups remained virtually unchanged after MCMV infection (**Figures 2C**, **S3A, C, D**).

In CD8 T cell (*Cd8*+, *Klrb1c*-), we defined the T07 cluster as CD8 naïve T cell (Tn) for low expression of *Cd44* and high expression of *Cd62l* (**Figures S3A, D**). For NKT cells (T03, T04, T06, T08, T13), based on the expression of *Cd62l*, we defined three groups of central memory NKT cells (Tcm-like, *Cd62l*+) that included T04 (ISG+CD8 NKT cell, *Ccr7*-), T06 (*Igfbp7* high, *Ccr7*+), and T13 (*Cd79a* high, *Ccr7*+) (**Figure S3D**). In *Cd62l* low cluster, we defined two groups of *Cxcr6*+ effector tissue resident NKT cells (Trm like *Cd62l* low, *Ccr7*-) that included T03 (*Cd103*-) and T08 (*Trdc* high) (**Figure S3D**). It was a remarkable discovery that the predominant NKT cell in the liver of infant mice was CD8αα+, which had the prominent ability to produce anti-viral cytokines (**Figures S3A, E**).

In this study, T01 cells (*Cd8*+, *Klrb1c*+*, Cd44*+, *Cd62l*+) highly expressed the proliferation marker *Mki67*, the tissue resident marker *Cxcr6*, *Cd69*, and immune effector genes *Ifn-γ*, *Gzmb*, *Perforin*(*Prf1*), which showed central memory phenotype (**Figures 2F**, **S3A, D, F**). Next, we traced the cell fate and reconstructed the cell lineage direction using the recently developed RNA velocity approach (23). We observed that the effector cytotoxic CD8+ T cells in T01 were predicted to mainly derive from T07 CD8+ Tn cells (**Figure 2D**). Furthermore, we used Monocle to place these populations along possible trajectories in pseudo-time (**Figure S3G**) and achieved the same results as those with RNA velocity analysis (**Figure 2D**). It is worth noting that our study consists of a limited number of samples (one sample per group), these findings only provide an evidence supporting the notion that T07 CD8+ Tn cells contribute significantly to the generation of T01 effector cytotoxic CD8+ T cells. T01 was found to be the most abundant group, and the percentage of T01 cells changed mostly during infection (**Figures 2C**, **S3C, K**). The Gene Ontology(GO) enrichment analysis also displayed the enriched terms for “cell cycle” and “nuclear division”, compared with that for other subclusters (**Figure 2E**). Using CD8 and MKI67 as markers, immunofluorescence staining and flow cytometry was performed and a significant increase in the number of *Cd8*+ *Mki67*+ cells after CMV infection was observed (**Figures 2F–H**). Furthermore, T01 cells showed an NK phenotype with high expression of *Klrc2* after MCMV infection (**Figure S3H**). According to a previous report, expanded CD8+T cells in the peripheral blood of HCMV-seropositive adults showed an NK phenotype and a broad immune surveillance capacity (24). In this study, the T01 proliferating CD8+ T cells were first identified, which existed specifically in the liver of infant mice with highly expressed resident markers, responded to MCMV infection, and showed an NK phenotype (**Figures S3D, H, J**). The liver resident T01 proliferating CD8+ T cells showed increased expression of of NK like marker: *Klrc2*, *Bcl11b* and exhaustion marker: *Pdcd1*(*Pd-1*), *Lag3* after MCMV infection (**Figures S3H, I**); these T cells differ from the NKG2C+CD8+ T cells detected in the peripheral blood of adults (24). Furthermore, T01 was segregated into 3 sub-clusters and identified as T01-1 NK (*Mki67* high, *Klrg1* high, *Cd8a*-, *Cd8b*-), T01-2 proliferating CD8 1(*Mki67* high, *Klrg1*-, *Plk1*+, *Cd8a*+, *Cd8b*+), and T01-3 proliferating CD8 2(*Mki67* high, *Klrg1*-, *Plk1*-, *Cd8a*+, *Cd8b*-) (**Figure 2I**). Further investigations of their detailed functions are yet to be conducted.

Besides T01, T04 ISG+CD8 NKT cells also increased in percentage after MCMV infection (**Figures 2C**, **S3C, K**). In addition to a subset of ISGs, T04 ISG+CD8 NKT cells also expressed high levels of *Cd62l* and *Cd44*; however, *Ccr7* expression was low (**Figures 2F**, **S3B, D, E**). These results indicated that the tissue resident ISG+CD8 T cells were activated after CMV infection and expressed various anti-viral cytokines. In a previous report, *Klrg1*+ *Cd27*+ cells demonstrated robust cytotoxicity against viral infection (25). It was also found that T12 cNK (*Klrg1*+ *Cd27*+ *Cd127*-) expressed high levels of cytotoxic genes (*Ifnγ*, *Gzmb* and *Perforin*) and low levels of ISGs (**Figures S3E, F**), proving its cytotoxic role in MCMV-infected livers.

Finally, we defined cluster T06 as a Tcm like NKT (*Igfbp7* high), which was similar to T04 ISG+CD8 T cells with respect to high ISG production abilities; It may also play an important role against MCMV infection (**Figures S3E, F**).

### Single-cell RNA sequencing identified a subset of monocytes induced by MCMV infection in the liver of infant mice

Monocytes are precursors for inflammatory macrophages, inflammatory monocytes, and dendritic cells, and play key roles in homeostasis, anti-pathogen response, and inflammation (9). Expression of *Clec4f* was lower with monocytes than with hepatic macrophages and dendritic cells (DCs) (**Figure S4A**). Based on previous reports, monocytes were classified into 3 major subgroups, classical (*Ly6c* high) monocyte, intermediate (*Cd43* high, *Ly6c* high) monocyte, and non-classical (*Ly6c* low) monocyte (**Figure S4B**) (26, 27). Classical (*Ly6c* high) monocytes showed an inflammatory phenotype with high expression of *Nos2* and multiple chemokines (*Ccl7*, *Ccl12*) (**Figure S4E**). Intermediate (*Cd43* high, *Ly6c* high) monocytes expressed high levels of *Mki67*, show proliferating phenotype (**Figure S4K**); they also highly expressed precursor monocyte marker *Ms4a3* per a previous report (28). Non-classical monocytes showed high *Cx3cr1* expression and had low migration capacity (*Ccr2* low) (**Figure S4B**).

**Figure 3:**
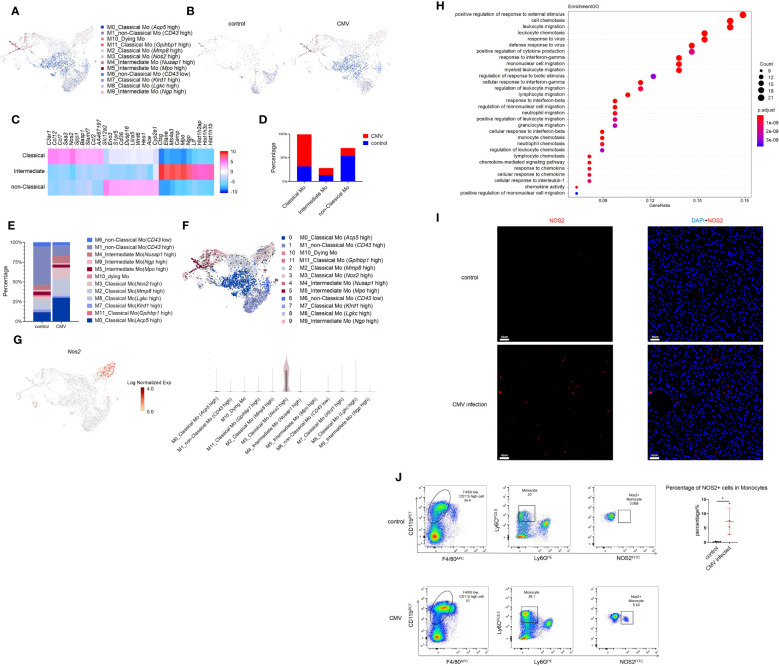
Single-cell RNA sequencing identifies a subset of Monocyte induced by MCMV infection in infant mice liver. **(A)** The Monocyte cell cluster in **Figure 1A** were re-clustered. UMAP of combined sub-clustered Monocyte cells from uninfected and infected infant mice liver. **(B)** UMAPs of 1,065 Monocytes from uninfected mice versus 3,074 Monocytes from infected mice. **(C)** Marker genes of main clusters in monocytes of infant mince liver. **(D)** The quantified percentage of three main Monocyte clusters between uninfected and infected mice liver. **(E)** The quantified percentage of each sub-cluster between uninfected and infected mice liver. **(F)** RNA velocities in scRNA-seq data of combined monocyte sub-clusters derived from control and infected mice liver. **(G)** Distribution and expression of MCMV response marker *Nos2* in combined sub-clustered Monocyte cells from uninfected and infected infant mice liver. **(H)** Enriched GO terms for significantly up-regulated genes in cluster classical monocyte(*Nos2* high), compared with other clusters. **(I)** Immunofluorescent labeling of NOS2+ cells (red) in uninfected and infected mice liver (Scale bars, 60 µm.). Cells nuclei were visualized with 4,6-diamidino-2-phenylindole (DAPI, Invitrogen). Experiments in **(I)** were repeated twice. **(J)** Representative flow cytometry (FC) plots and quantification showing the percentage of NOS2+ cells in Monocytes in the liver from indicated mice (n = 4, mean ± SD). Statistical significance was determined by non-parametric Mann Whitney test between groups (*P < 0.05). Experiments were repeated two times.

Monocytes increased significantly in the liver of infant mice and were characterized primarily by classical monocytes during CMV infection (**Figures 3A–D**). Classical monocytes were further divided into six groups; of these, classical Mo (*Acp5* high), classical Mo (*Mmp8* high), and classical Mo (*Nos2* high) were predominant. These clusters increased significantly after infection (**Figures 3E**, **S4C–E, L**).

According to RNA velocity analysis, it’s suggested that classical monocytes may predominantly differentiate into Cluster 3 classical monocyte (Nos2 high) (**Figures 3F, G**). The GO analysis on this cluster showed its strong antiviral and migratory ability (**Figure 3H**). Immunofluorescence staining was performed to verify the findings at the protein level. Since *Nos2* was a specific marker for this cell group (**Figures 3G**, **S4F**), a comparison between the infected and non-infected livers showed a significant increase in Nos2+ cells after infection (**Figure 3I**). Furthermore, flow cytometry was performed for validation, and it yielded consistent conclusions, a notable increase in NOS2+ monocytes was observed post-infection. (**Figure 3J**). Next, pseudo-time analysis was performed on all monocytes and combined with RNA velocity analysis (**Figure 3F**). It was found that the branching of monocytes at node 1 was caused by MCMV infection (**Figures S4G**, **H**). On further analysis of node 1, it was predicted that cell fate 1 and 2 preferentially differentiated into non-classical Mo (*CD43* high) and classical monocyte (*Nos2* high), respectively. Compared with other cells, the expression of *Cxcr4*, *Cytip* and other adhesion-related genes (*Fn1*, *Tagln2*) in cell fate 2 decreased gradually, while the expression of Interferon-induced proteins (*Ifi203*, *Ifi204*, *Ifi205*, *Ifi207*, *Ifi209*, *Ifi211*) related to immune activation increased gradually (**Figure S4I**). Given the limited number of samples (one per group), these results based on trajectory inference must be taken with caution and further research and validation is required to draw definitive conclusions. The GO analysis on Cluster 3 classical monocytes (*Nos2* high) between the infected and the control groups was also performed, which showed a significant increase in the antiviral function in Cluster 3 cells after MCMV infection (**Figure S4J**). In general, a population of monocytes was found that increased specifically after MCMV infection. and may have acted as terminal effector cells and played an anti-viral role.

### MCMV infection interfered with the PPAR-γ pathway and altered protein expression of lipid metabolism in infant mice hepatocytes

The liver is a key metabolic organ governing energy metabolism, which could be interrupted by HCMV infection (29, 30). Hepatic lipase (Lipc, Hl) and lecithin-cholesterol acyltransferase (Lcat) are considered to be the key liver enzymes of lipid metabolism. The content and activity of HL and LCAT were significantly decreased in HCMV-IgG+ patients (31). Low-density lipoprotein receptor-related protein 1 (Lrp1), which controls lipid homeostasis, was increased during early CMV infection, resulting in lowered intracellular cholesterol levels (32). Results were consistent with our scRNA-seq data, which further reinforced the impact of CMV infection on host liver lipid metabolism (**Figure S5A**).

**Figure 4:**
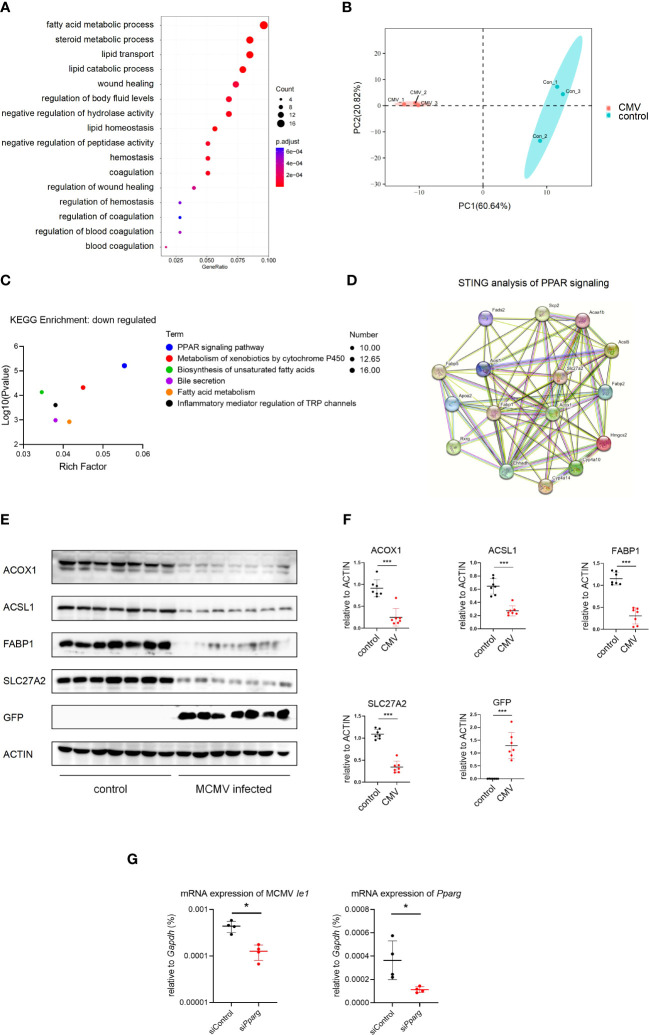
MCMV infection influence the lipid metabolism pathway in infant mice hepatocytes. **(A)** The hepatocytes cell cluster in **Figure 1A** were selected. Enriched GO terms for significantly down-regulated genes in hepatocytes cluster, compared with other clusters, were shown as bubble diagram. **(B)** Hepatocytes from three uninfected and three MCMV infected 2 weeks old WT mice were isolated and proteomic mass spectrometry analysis were performed individually. PCA analysis showed that the difference between infection group and control group. Each point represents an individual mice. **(C)** Enriched KEGG terms for significantly down-regulated genes of hepatocytes between uninfected and infected group in proteomic mass spectrometry analysis. **(D)** Proteomic gene regulation network of down-regulated PPAR-γ pathway analyzed by STRING. **(E)** WT mice were infected by MCMV at 14 days post birth. Immunoblotting with antibodies target ACOX1, ACSL1, FABP1, SLC27A2 and GFP in hepatocytes from mice infected with MCMV at three dpi or treated with PBS. Experiments were repeated three times. Each lane represents an individual mice. **(F)** Quantitative analysis of **(E)** (mean ± SD). Statistical significance was determined by non-parametric Mann Whitney test between groups (*P < 0.05, ***P < 0.001). **(G)** AML12 mice hepatocyte cell were transfected with siRNA that does not match any known genes or regulatory regions(siControl) or target *Pparg* mRNA(si*Pparg*). qPCR analysis of indicated genes in different cells at 24 hours post MCMV infection (n=4, mean ± SD). Statistical significance was determined by non-parametric Mann Whitney test between groups (*P < 0.05, ***P < 0.001). Experiments were repeated three times. Each point represents an individual cell sample.

Hepatocytes constitute approximately 80% of the liver and play an important role in lipid metabolism (10). To investigate the effect of CMV infection on infant mice hepatocytes, GO analysis of hepatocytes was performed between the infected and control groups using our scRNA-seq data. It was found that CMV infection significantly enhanced anti-viral response and decreased gene expression related to lipid metabolism (**Figures 4A**, **S5B**). We further performed proteomic analysis of the isolated primary hepatocytes before and after infection. The difference between the infection group and the control group was shown by principal component analysis (PCA) analysis (**Figure 4B**), which was further confirmed by GO and Kyoto Encyclopedia of Genes and Genomes (KEGG) analyses of the proteome (**Figures 4C**, **S5B–D**). Peroxisome proliferator-activated receptors (PPAR) signaling is an important pathway that controls whole-body nutrient/energy homeostasis in the liver (33). We utilized STRING to analyze differentially expressed genes within the PPAR signaling network, and the resulting interaction network is visually depicted (**Figure 4D**). This representation simplifies the understanding of intricate relationships among the downregulated genes in the PPAR pathway, thereby providing a more comprehensive understanding of the gene interconnections in hepatocytes following CMV infection. Furthermore, we identified four proteins, ACOX1, ACSL1, FABP1, and SLC27A2, located at the central part of this network, and subsequently performed Western blot analysis to confirm the downregulation of their expression levels (**Figures 4E, F**). We continued to investigate the impact of PPAR-γ on CMV infection in hepatocytes. Interestingly, knockdown of PPAR-γ in hepatocytes resulted in the inhibition of CMV infection (**Figure 4G**).

## Discussion

Since CMV is highly virulent, most adults are prone to infection. Previous studies have demonstrated an immune response against CMV infection in adults (1, 21). However, the immune system matures gradually during development (34). The compromised immune system in newborns predisposes them to CMV infection, resulting in serious diseases (19). Congenital and postnatal CMV infection leads to changes in infant CMV-specific CD4 and CD8 T cells and the overall T cell population (35). There is increased activation of the entire CD8 T cell population, which normalizes within 12-24 months (36). However, these observations are based on the detection of major cell populations, and it is crucial to employ higher-resolution single-cell profiling to gain a more detailed understanding. Moreover, obtaining suitable human samples during the early stages of CMV infection in infants is challenging. Nevertheless, studying the early immune response against CMV is essential for a comprehensive understanding of the immune system and the intricate interplay between the virus and the host. Utilizing mouse models provides a viable alternative for investigating these aspects, allowing us to delve deeper into the mechanisms and gain valuable insights.

The liver is the primary site of systemic CMV infection (1). Using single-cell RNA-seq, the alteration of immune cells in liver after MCMV infection was clearly depicted. We clarified that the liver resident T01 proliferating CD8 T cells responded to the first early-stage CMV exposure in infant mice, showing its strong anti-viral ability. T01 showed characteristics of both NK and T cells, with higher expression of the exhaustion marker after virus infection. The study also identified a cluster of monocytes in the liver of infant mice with high NOS2 expression, which may play an important role in the early anti-viral process. In addition, it was observed that MCMV infection altered protein expression of lipid metabolism and interfered with the PPAR-γpathway in hepatocytes.

Previous studies have shown interactions between CMV infection and lipid metabolism, HCMV leverages a host stress response to balance the elongation of saturated/monounsaturated and polyunsaturated Very-Long-Chain Fatty Acids, a process crucial for virus infection in fibroblasts (37). Another research noted that in neuronal cells, CMV infection enhances the expression of PPAR-γ, further facilitating CMV infection (38). In the current investigation, we provide the first *in vivo* evidence of alterations in the lipid metabolism-associated protein levels within murine hepatocytes during cytomegalovirus (CMV) infection, affirming the significant interplay between CMV infection and lipid metabolism. It was discerned that hepatocytes within the infected liver demonstrated a downregulation of the PPAR-γ pathway. Intriguingly, knocking down PPAR-γ expression in hepatocytes proved inhibitory for CMV infection. Considering the lower infection rates *in vivo* (**Figures 1E, F**) and the intricate cellular interactions within the hepatic milieu, this hepatocyte-specific alteration might be interpreted as a stress response elicited by viral infection, or alternatively, as a hepatocytic reaction to intercellular signaling, which could conversely curb viral infection. Future research endeavors should aim to comprehensively dissect the intricate relationship between lipidomic profiles and immune responses, and specifically delineate the alterations within infantile hepatic lipid metabolism concurrent with CMV infection.

## Materials and methods

### Mice

The wild type C57BL/6 male mice were purchased from GemPharmatech. Mice were maintained in SPF conditions under a strict 12 hr light cycle (lights on at 08:00 and off at 20:00). All animal studies were performed according to approved protocols by the Ethics Committee at the University of Science and Technology of China.

### Cell culture

The AML12 mice hepatocyte cell line (Hycyte™, TCM-C709) was obtained from the Suzhou Haixing Biosciences Co., Ltd. Cells were cultured in AML12 cell-specific culture medium(Hycyte™, TCM-G709, DMEM/F12 medium+10% fetal bovine serum (FBS)+1%ITS+40ng/mL Dexamethasone+1%P/S). All cells were cultured at 37°C in 5% CO2 using culture dishes purchased from Guangzhou Jet Bio-Filtration Co., Ltd. All cells were tested for eliminating the possibility of mycoplasma contamination.

### Virus

MCMV-smith (provided by Prof. Daxing Gao (University of Science and Technology of China)) was propagated on C57BL/6J primary mouse embryonic fibroblasts (MEFs), and viral titers were determined using a 96-well plate (Guangzhou Jet Bio-Filtration Co., Ltd). Briefly, cell-free media from the virus-infected cultures were collected to determine the virus titers using the 50% tissue culture infectious dose (TCID50) assay in MEFs. Experimental infant mice (2 weeks) were infected with MCMV by intraperitoneal injection of 5×10^6^ TCID50 in 100 ul DMEM.

### RNA interference

Indicated siRNA targeting mice *Pparg* and control siRNA(siControl) were transfected into AML12 cell using Lipofectamine 3000 (Invitrogen, Carlsbad, CA, USA) according to the manufacturer’s instructions. After 48 hours of transfection, cells were used for further experiments.

The siRNA sequences are listed below:

si*Pparg*-Sense: CGCAUUCCUUUGACAUCAA(dT)(dT),
si*Pparg*-Anti-Sense: UUGAUGUCAAAGGAAUGCG(dT)(dT),
siControl-Sense : UUCUCCGAACGUGUCACGU(dT)(dT),
siControl-Anti-sense:ACGUGACACGUUCGGAGAA(dT)(dT).

### Hepatocyte collection

Mouse liver were perfused with EGTA solution and digested with 0.075% (m/v) type I collagenase (BS163, biosharp) at 37°C. Isolated cells were resuspended in serum-free Dulbecco’s modified Eagle’s medium (SH30022.01; Hyclone, Logan, USA) and layered onto 60% (v/v) Percoll solution (17-0891-09; Cytiva, Logan, USA), followed by centrifugation at 400×g and collection of hepatocytes.

### Proteome analysis

Hepatocytes were isolated and frozed in liquid nitrogen immediately. The protein of samples frozed in liquid nitrogen was extracted by lysis buffer containing 1 mM PMSF and 2 mM EDTA. Samples digested with trypsin was labeled with TMT (Thermo Scientific) Label Reagent Set. The labeled samples were fractionated by high-performance liquid chromatography (HPLC) system (Thermo DINOEX Ultimate 3000 BioRS) using a Welch C18 column and analyzed using Q Exactive plus mass spectrometer (Thermo Fisher) coupled with the UltiMate 3000 RSLC nano system (Thermo Fisher). We obtained quantitative values for each sample through quantitative analysis using Maxquant 1.6.17.0 software. After normalizing the quantification values within and between samples, we calculate the fold change in expression between the two groups of samples for each comparison group. The criteria for determining significant differential expression are as follows: when adj.P-value ≤ 0.05 and the fold change ≥ 1.5 (downregulated expression) or fold change ≤ 0.667 (upregulated expression), it is considered to be a significant expression change. Differential protein sequences were compared with the NR database using Blast-2.6.0 (BlastT). NR annotation results were then used in the Gene Ontology database (2023-04-08 release, http://geneontology.org/) and KEGG database (March 6, 2023, https://www.kegg.jp) for functional annotation analysis.

### Flow-cytometric assays

Liver immune cells were isolated from experimental mice by forcing the dissected liver through 200-G stainless steel mesh. Then, immune cells were isolated by gradient centrifugation with 40% Percoll™ solutions and the subsequent lysing of erythrocytes (Red Cell lysis Solution, Biosharp, BL503A). After blockade of Fc receptors with CD16/32 (Biolegend, 101302), cells were stained with fluorescence-conjugated antibodies (CD11b-PE (Biolegend, 101208), Ly6G-FITC (Biolegend, 127606), F4/80-APC (Biolegend, 123116), Ly-6C-BV510 (Biolegend, 128033), CD11c-PE/Cy7 (Biolegend, 117318), CD19-APC/Cy7 (Biolegend, 115530), CD3-PE (Biolegend, 100206), CD4-Super Bright 600 (Invitrogen, 63-0042-82), CD8a-PE/Cy7 (Biolegend, 100722), NK-1.1-APC (Biolegend, 108710)), Fixable Viability Dye (Biolegend, 423102), CD3-FITC (Biolegend, 100204), CD4-APC (Biolegend, 100412), Ly6G-PE (Biolegend, 127608), Ly-6C-PerCP-Cy5.5 (Biolegend, 128012), CD11b-PE/Cy7 (Biolegend, 101216). For intracellular cytokine staining (Ki67 and iNOS), 2x10^6^ cells pre-stained with appropriate surface marker antibodies were fixed/permeabilized for 60 min at 4°C with Transcription Factor Buffer Set (BD Pharmingen, 562574) and stained with fluorescently-conjugated iNOS-FITC (Invitrogen, 53-5920-80) or Ki67-PE (Biolegend, 652404) antibodies per manufacture’s protocols. After wash, cells were analyzed using CytoFLEXS (BECKMAN COULTER). Flow cytometry data were analyzed using FlowJo v.10. The staining antibodies for flow cytometry all used at 1:200 unless otherwise indicated.

### RT-qPCR

For cells, total RNA was extracted with TRNzol Universal reagent (Tiangen) in accordance with the manufacturer’s instructions. Real-time PCR was performed using SYBR Premix Ex Taq II (Tli RNaseH Plus) (Takara) and complementary DNA was synthesized with a PrimeScript RT reagent Kit with gDNA Eraser (Takara). The target genes were normalized to the housekeeping gene (*Gapdh*) shown as 2^−ΔCt^. The used primers target mice gene are as follows:

*Gapdh*-F: TGAGGCCGGTGCTGAGTATGTCG

*Gapdh*-R: CCACAGTCTTCTGGGTGGCAGTG

*Pparg*-F: TCGCTGATGCACTGCCTATG

*Pparg*-R: GAGAGGTCCACAGAGCTGATT

MCMV-*Ie1*-F: AGCCACCAACATTGACCACGCAC

MCMV-*Ie1*-R: GCCCCAACCAGGACACACAACTC

### Western blot

Isolated Hepatocytes were lysed with RIPA buffer (Beyotime Biotechnology) supplemented with PMSF (Beyotime Biotechnology). ACOX1(Proteintech, 10957-1-AP), ACSL1(Proteintech, 13989-1-AP), FABP1(Bioswamp, PAB33952), SLC27A2(Bioswamp, PAB30568), GFP(Boster, BM3883), ACTIN(Proteintech, 66009-1-Ig) antibodies were used in accordance with the manufacturer’s instructions. After incubation with the primary antibody overnight, the blotted PVDF membranes (Immobilon, IPVH00010) were incubated with goat anti-rabbit IgG-HRP (Beyotime, A0208) or goat anti-mouse IgG-HRP (Beyotime, A0216) and exposed with BIO-RAD ChemiDocTM Imaging System for a proper exposure period.

### Immunofluorescent staining

Livers were isolated from wild-type C57BL/6J mice infected by CMV, as well as the wild-type littermate control. The separated liver was immersed in EDTA antigen retrieval buffer (pH 8.0). 3% BSA were added to cover the tissue to block non-specific binding for 30 min. The primary antibody (Anti-iNOS Rabbit pAb, GB13495, Servicebio; Anti-Ki67 Mouse mAb, GB121142, Servicebio; Anti-CD8 alpha Rabbit pAb, GB114196, Servicebio) was incubated at 4°C overnight, followed by incubation with Secondary antibody for 50min at room temperature. The cell nuclei were counterstained with 4,6-diamidino-2-phenylindole (DAPI, Invitrogen) and incubated for 10min at room temperature under light. The autofluorescence quencher were added for 5min and rinse with water for 10min. The slices were slightly dried and sealed with anti-fluorescence quenched tablet. All fluorescence images were analyzed using Nikon DS-U3 confocal imaging.

### 10x genomics single-cell transcriptome

Liver tissue was dissociated for single cell RNA sequencing. Viability enrichment was performed using the Dead Cell Removal Kit (130-090-101, Miltenyi Biotec) as manufacturers protocol. The cells were resuscitated to a concentration of 700-1200 cells/ul (viability ≥ 85%) in a final solution of 1 x PBS + 0.04% BSA prior to loading on the 10×Genomics Chromium platform. 10,000 cells were used to prepare scRNA-seq libraries. Chromium Single cell 3’ Library and Gel Bead Kit V3.1 (10× Genomics, PN1000268) was used to generate single cell gel beads in emulsion (GEM). After quality control, the libraries were sequenced on Illumina Novaseq 6000 platform in 150 bp pair-ended manner (Berry Genomics Corporation, Beijing, China). The software used for subsequent analysis is listed in **Table 1**.

**Table 1:** Software used in the study.

Software	Version	Description	
Cell Ranger	4.0	10x Genomics official scRNA-seq data analysis software	https://support.10xgenomics.com/single-cell-gene-expression/software/pipelines/latest/using/tutorial_in
Seurat	3.1	Data analysis software	https://satijalab.org/seurat/
Monocle	2.0	Pseudotime analysis software	http://cole-trapnell-lab.github.io/monocle-release/docs/
SingleR	1.0.5	Automatic cell type annotation software	https://github.com/dviraran/SingleR
Velocyto	0.17.17	Generating loom file	velocyto.org
Scrublet	0.2.1	Doublet cell prediction software	https://github.com/swolock/scrublet
R	3.5.1	Statistical computing software	https://cran.r-project.org/
scVelo	0.2.3	Cell velocity analysis software	https://scvelo.readthedocs.io/en/stable/

### Quality control

Raw data (Raw Reads) of fastq files were assembled from the Raw BCL files using Illumina’s bcl2fastq converter. For Raw data, firstly processed through primary quality control. The monitored quality assessment parameters were, (i) contain N more than 3; (ii) the proportion of base with quality value below 5 is more than 20%; (iii) adapter sequence. All the downstream analyses were based on the clean data with high quality.

### Single-cell transcriptome data processing

All of single-cell transcriptome sequencing data were aligned and quantified by Cell Ranger (V6.0.2, Linux) against the reference genome from 10x Genomics official website, and default parameters. All downstream single-cell analyses were performed using Cell Ranger and Seurat unless mentioned specifically.

Cellranger count takes FASTQ files performs alignment, filtering, Barcode counting, and UMI counting. It uses the Chromium cellular Barcodes to generate feature Barcode matrices by cellranger count or cellranger aggr and reruns the dimensionality reduction, clustering, and gene expression algorithms using cellranger default parameter settings.

The preliminary counts were then used for downstream data analysis by Seurat (V3.1.5, R), including quality control, normalization, feature selection, dimension reduction, unsupervised clustering, differential expression, and visualization. Quality control was performed to remove the low-quality cells, with less than 200 detected genes, fewer than 400 UMI countsor more than 20% mitochondrial gene counts. Then genes expressed in less than 3 cells were also removed. Finally, Scrublet v.0.2.1 was used to assess and remove the effect of droplets that may have contained more than one cell, with an expected doublet rate of 0.07 and a score threshold of 0.25(multiplets cell number: control: 651; CMV: 418)(removed cell number: control: 3189; CMV: 1143) (remaining cell number: 16699; CMV: 16658).

### Unsupervised clustering analysis

The preprocessed data were normalized and scaled using Seurat function NormalizeData, and FindVariableFeatures (Seurat) was used to identify highly variable genes. By default, we chose the top 2000 variable features genes for subsequent dimensionality reduction and cell clustering analysis. The principal components (PCs=30) were estimated by RunPCA. UMAP and t-SNE dimensionality reduction were then performed by the RunUMAP or RunTSNE to place cells and visualize the distribution of cells. Seurat function FindIntegrationAnchors and IntegrateData were used to remove batch effect from different batches and integrate single cell data. Next, we applied CellMarker V2.0 database to finish the cell type recognition via singleR 1.0.5 (39). Further verification of cell types and subtypes was completed according to their expression of the known canonical marker genes of the respective cell types, for example, *Vpreb3*, *Gzma*, and *Ptprb* were used to identify B cells, T cells and endothelial cells, respectively (40, 41).

### Differential gene expression and pathways enrichment analyses

In order to identify signature genes in each cell type, Seurat function FindAllMarkers were carried out (min.pct =0.25, test.use =‘wilcox’, return.thresh =0.25) to identify differentially expressed genes (DEGs). To annotate the function of these DEGs, we performed GO enrichment analysis of differentially expressed gene sets were implemented in the GOseq R. GO terms with adjusted P-value below 0.05 were considered as significantly enriched by differential expressed genes.

### Trajectory analysis by Monocle 2

We performed the trajectory analysis using pseudotime inferencing algorithm Monocle 2 to reconstruct the cell differentiation trajectory of different cell sub-clusters with default parameters (http://cole-trapnell-lab.github.io/monocle-release/docs/).

The Monocle package was used to plot trajectories to illustrate the behavioral similarity and transitions. It’ s used an expression matrix derived from Seurat to build a CellDataSet for Monocle pipeline, and partition the cells into supergroups after dimensionality reduction. OrderCells function was applied in organizing supergroups into a tree-like trajectory. Plot cell trajectory module was used to plot the trajectory and color the cells by sub-cluster type.

### RNA velocity analysis

To analyze RNA velocity, we utilized the Velocyto (v0.17.17) and Scvelo (0.2.3) packages. First, Velocyto was used to create a loom file from 10x genomics data with reference transcriptomes. We then read and prepared the data for analysis using Scvelo in an Anndata object. The dataset was normalized and filtered to ensure a minimum of shared counts and selection of the top genes. Moments were computed to capture dependencies and correlations of the data using principal component analysis (PCA) and neighborhood graphs. A dynamical model was then built by recovering the dynamics, calculating the RNA velocity and generating a velocity graph.

### CellCycleScoring

Cell Cycle Scoring function was used to assess the cell cycle stage of individual cells based on gene expression patterns with default settings following the instruction (https://satijalab.org/seurat/articles/cell_cycle_vignette.html).

### AddmoduleScore

AddModuleScore function was used to identify the cell responding to the signature pattern with default settings (features = marker gene, ctrl = 80) following the instruction (https://satijalab.org/seurat/reference/addmodulescore).

### scDC analysis

The quantified percentage of each cluster were calculated by scDC package (42) with default settings following the instruction (https://sydneybiox.github.io/scDC/index.html). scDC_noClustering function were used with default settings (calCI = TRUE, calCI_method = c(“BCa”), nboot = 10000, conf_level = 0.95).

### Statistical analysis

Statistical analysis was performed with the GraphPad Prism 8.0 (GraphPad, Inc, USA). Exclusion criteria were pre-established (34, 43, 44). Experiments were independently repeated for indicated times listed in the figure legend. Representative data was exhibited as the means ± SD. Quantitative data was compared using non-parametric Mann Whitney test. P-values for every result were labeled on figures, P < 0.05 was reckoned as statistically significant (*P < 0.05, **P < 0.01, ***P < 0.001, NS., not significant).

## Data availability statement

The datasets presented in this study can be found in online repositories. The names of the repository/repositories and accession number(s) can be found here: GSE225309 (GEO) and PXD040842 (ProteomeXchange).

## Ethics statement

The animal study was approved by Ethics Committee at the University of Science and Technology of China. The study was conducted in accordance with the local legislation and institutional requirements.

## Author contributions

JG designed, performed and interpreted experiments. AW and XB analyzed single cell seq. JG wrote the manuscript. AW and WJ edited the manuscript. WJ supervised the project. All authors contributed to the article and approved the submitted version.

## Supplementary material

Supplementary Figure 1 Marker genes of clusters from **Figure 1A** **. (A)** Distribution and expression of representative marker genes of different clusters in **Figure 1A**. **(B)** The AddModuleScore function from Seurat was used to identify different clusters in **Figure 1A** that respond to the signature pattern in **Figure S1A**.

Supplementary Figure 2 Extended analysis identified the alteration of cells in MCMV infected mice liver. **(A)** Experimental infant mice (2 weeks) were infected with MCMV by intraperitoneal injection of 5×106 TCID50 in 100ul DMEM for 3 days. Representative flow cytometry (FC) plots and quantification showing the absolute numbers of assigned cells of liver in control and MCMV infected mice (n = 4, mean ± SD). Statistical significance was determined by non-parametric Mann Whitney test between groups (*P < 0.05, **P < 0.01, NS., not significant). Experiments were repeated two times. **(B)** The scDC analysis was conducted to statistically validate the alteration of cell percentage in scRNA-seq data.

Supplementary Figure 3 Characteristic of T cell sub-clusters in infant mice liver. **(A, B)** The T cell cluster in were re-clustered. Distribution and expression of representative genes of combined sub-clustered T cells from uninfected and infected mice. **(C)** The quantified percentage of each sub-cluster from uninfected and infected mice T cells. **(D)** Distribution and expression of representative genes of combined sub-clustered T cells from uninfected and infected mice. **(E)** Expression heat map of a subset of ISGs in T cell sub-cluster from uninfected and infected mice, as revealed by scRNA-seq (normalized data). **(F)** Distribution and expression of representative cytotoxic T cell marker genes. **(G)** Pseudotime analysis of T cell sub-clusters by monocle 2. **(H)** Distribution and expression of representative NK cell markers. **(I)** Distribution and expression of representative exhausted T cell markers. **(J)** Cell cycle scoring were conducted to view the proliferation status of each T cell sub-clusters. **(K)** The scDC analysis was conducted to statistically validate the alteration of cell percentage in scRNA-seq data.

Supplementary Figure 4 Characteristic of Monocytes subclusters. **(A)** Expression of kupffer cell marker *Clec4f* of 4 clusters of myeloid cells in infant mice liver. **(B)** Distribution and expression of representative genes of 4 clusters of myeloid cells in infant mice liver. **(C)** The quantified percentage of each sub-cluster within Monocytes between uninfected and infected mice liver. **(D)** Distribution and exprssion of marker genes of all sub-clusters within Monocytes in infant mice liver. **(E)** Heat map of marker genes in 6 classical sub-clusters within Monocytes. **(F)** Distribution of *Nos2* in main clusters of infant mice liver. **(G-H)** Pseudotime analysis of Monocyte cell sub-clusters in Monocyte cells from uninfected and infected infant mice liver by monocle2. **(I)** Representative gene expression heat map of two different cell fate in Monocyte analyzed by monocle2. **(J)** Cluster classical monocyte(*Nos2* high) was selected to perform GO analysis between uninfected and infected mice. Enriched GO terms for significantly up-regulated genes were shown as bubble diagram. **(K)** Cell cycle scoring were conducted to view the proliferation status of each Monocyte sub-clusters. **(L)** The scDC analysis was conducted to statistically validate the alteration of cell percentage in scRNA-seq data.

Supplementary Figure 5 Multi-omics analysis of MCMV infected mice liver. **(A)** Distribution and expression of representative enzymes of lipid metabolism in uninfected and infected mice liver, as shown by scRNA-seq data. **(B)** Enriched GO terms for significantly down-regulated genes of hepatocytes between uninfected and infected group in proteomic mass spectrometry analysis. **(C)** Enriched KEGG terms for significantly up-regulated genes of hepatocytes between uninfected and infected group in proteomic mass spectrometry analysis. **(D)** Enriched GO terms for significantly up-regulated genes of hepatocytes between uninfected and infected group in proteomic mass spectrometry analysis.
