# Supplementary figures and images for: Acute murine cytomegalovirus infection boosts cell-type specific response and lipid metabolism changes in the liver of infant mice
Source: Front Immunol. 2023 Aug 10;14:1169869. doi: 10.3389/fimmu.2023.1169869 (PMC10449610; doi:10.3389/fimmu.2023.1169869)

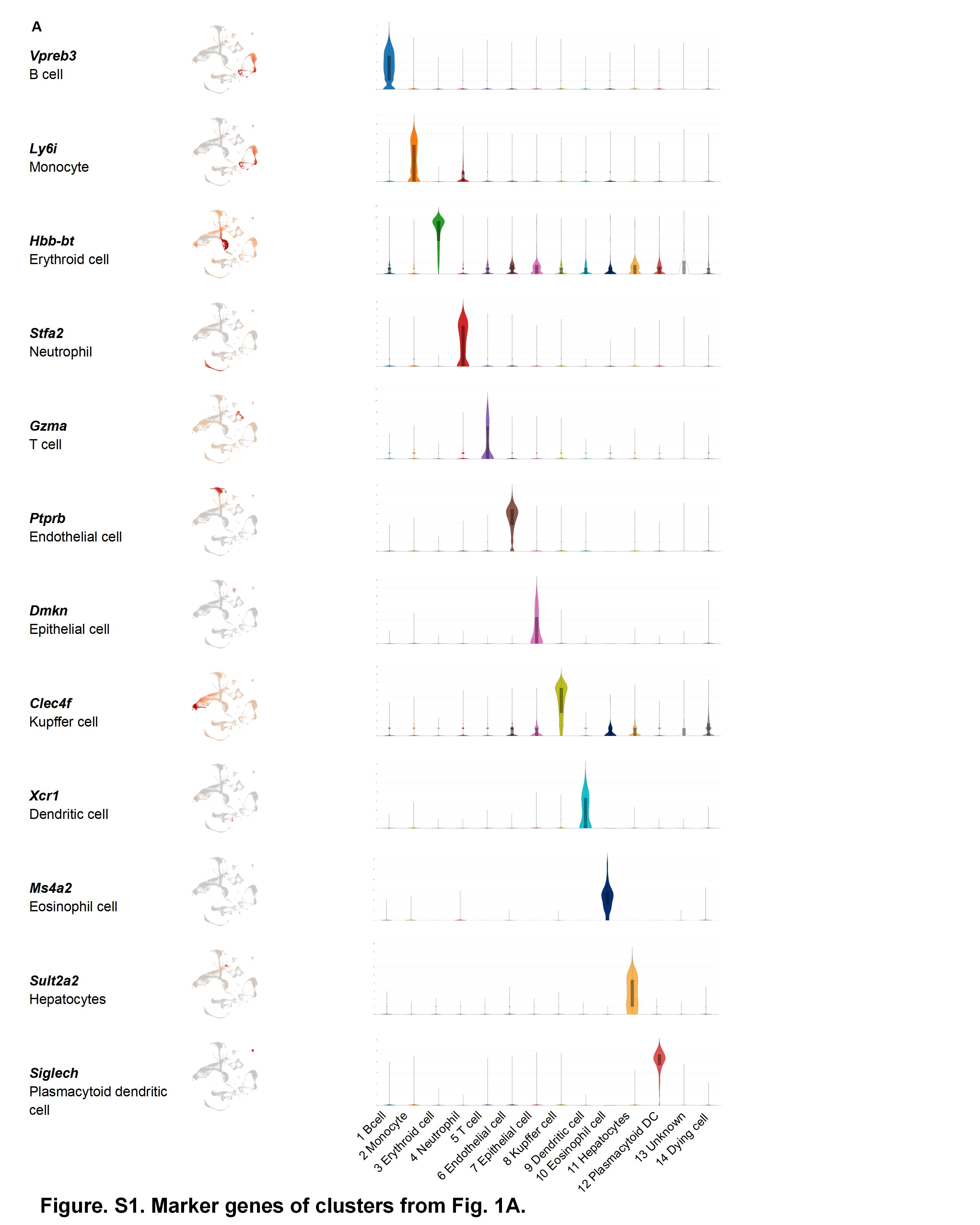

Supplement: Supplementary Figure 1 — Marker genes of clusters from Figure 1A . (A) Distribution and expression of representative marker genes of different clusters in Figure 1A . (B) The AddModuleScore function from Seurat was used to identify different clusters in Figure 1A that respond to the signature pattern in Figure S1A . [file Image_1.jpeg]

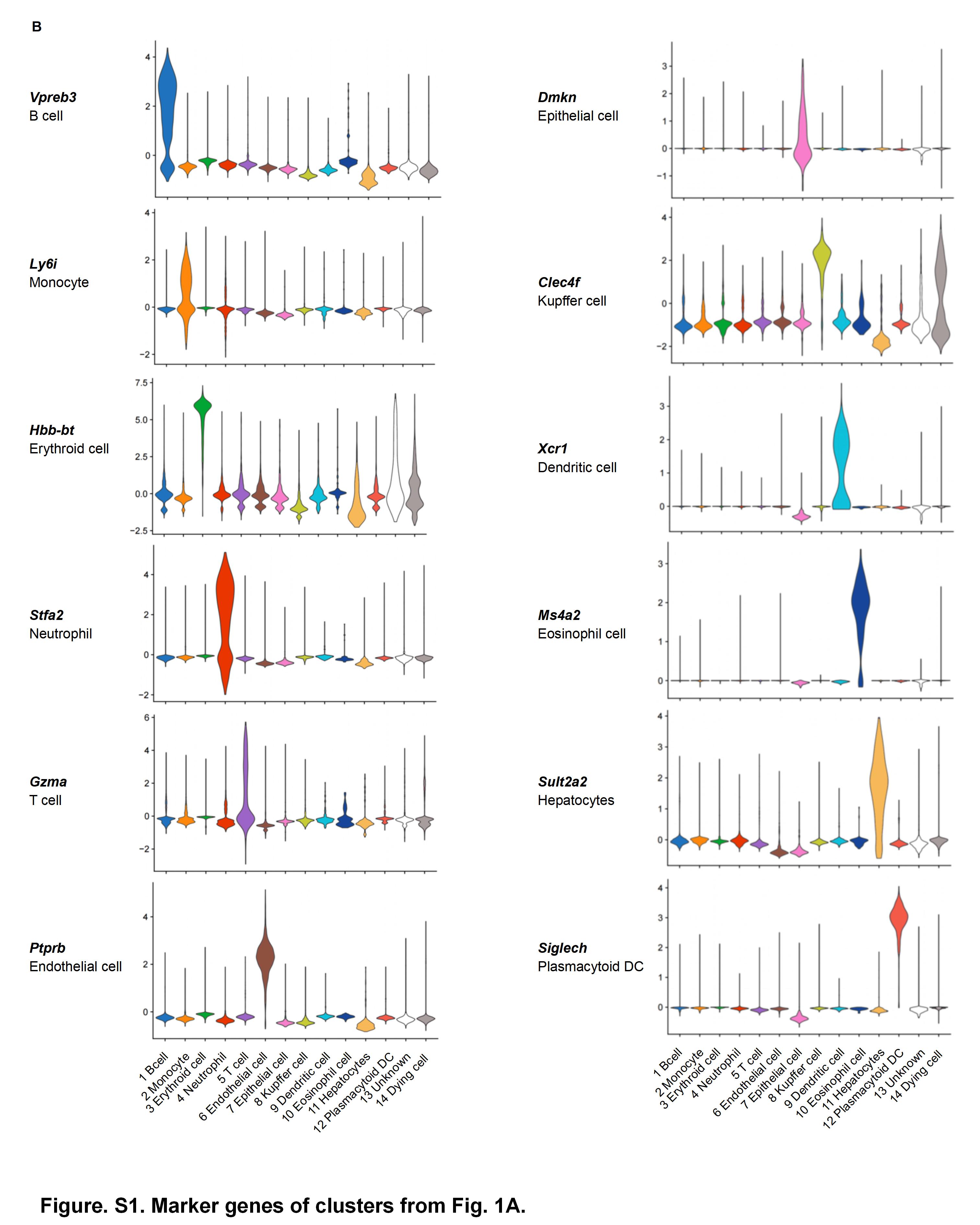

Supplement: Supplementary Figure 2 — Extended analysis identified the alteration of cells in MCMV infected mice liver. (A) Experimental infant mice (2 weeks) were infected with MCMV by intraperitoneal injection of 5×10^6^ TCID50 in 100ul DMEM for 3 days. Representative flow cytometry (FC) plots and quantification showing the absolute numbers of assigned cells of liver in control and MCMV infected mice (n = 4, mean ± SD). Statistical significance was determined by non-parametric Mann Whitney test between groups (*P < 0.05, **P < 0.01, NS., not significant). Experiments were repeated two times. (B) The scDC analysis was conducted to statistically validate the alteration of cell percentage in scRNA-seq data. [file Image_2.jpeg]

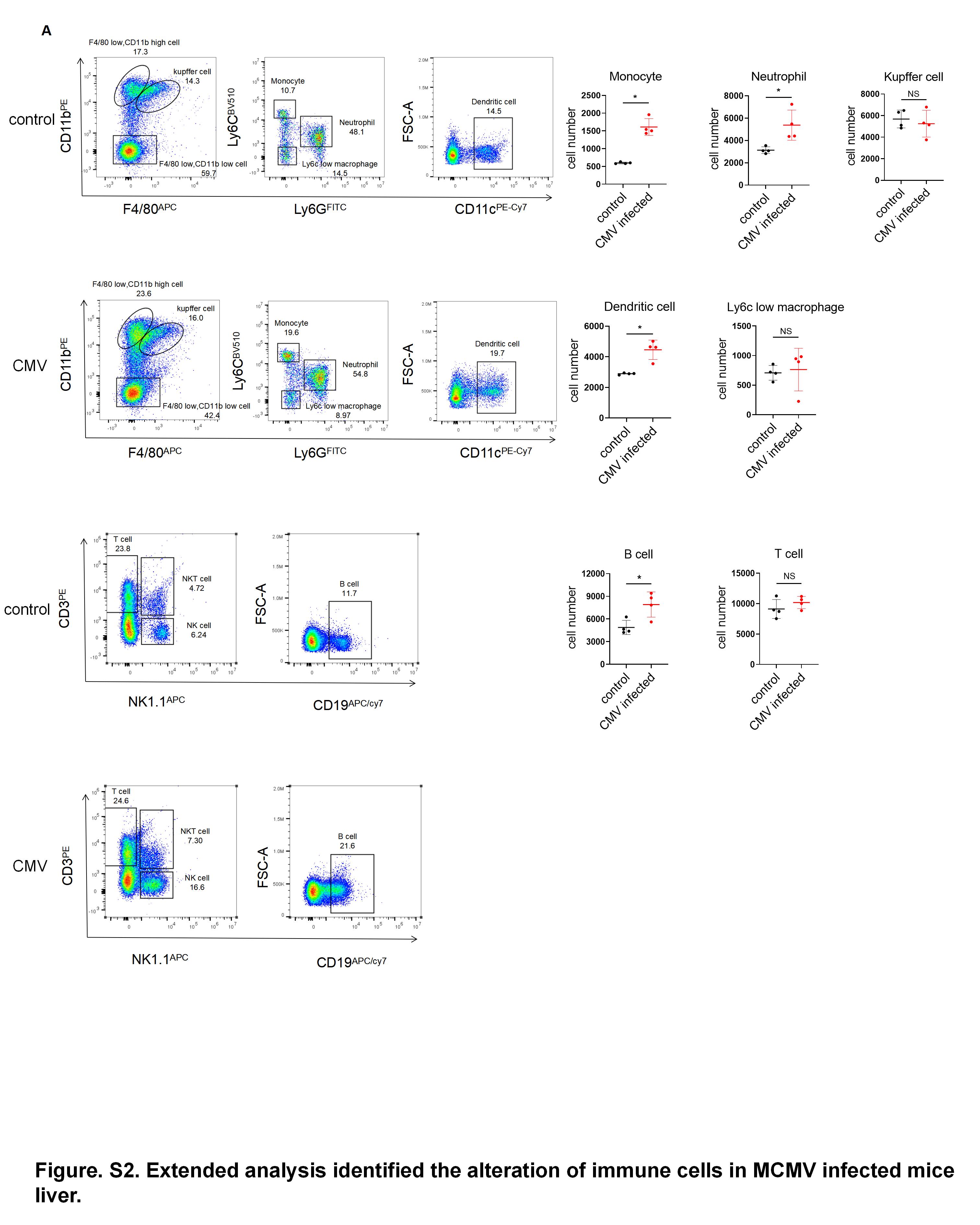

Supplement: Supplementary Figure 3 — Characteristic of T cell sub-clusters in infant mice liver. (A, B) The T cell cluster in were re-clustered. Distribution and expression of representative genes of combined sub-clustered T cells from uninfected and infected mice. (C) The quantified percentage of each sub-cluster from uninfected and infected mice T cells. (D) Distribution and expression of representative genes of combined sub-clustered T cells from uninfected and infected mice. (E) Expression heat map of a subset of ISGs in T cell sub-cluster from uninfected and infected mice, as revealed by scRNA-seq (normalized data). (F) Distribution and expression of representative cytotoxic T cell marker genes. (G) Pseudotime analysis of T cell sub-clusters by monocle 2. (H) Distribution and expression of representative NK cell markers. (I) Distribution and expression of representative exhausted T cell markers. (J) Cell cycle scoring were conducted to view the proliferation status of each T cell sub-clusters. (K) The scDC analysis was conducted to statistically validate the alteration of cell percentage in scRNA-seq data. [file Image_3.jpeg]

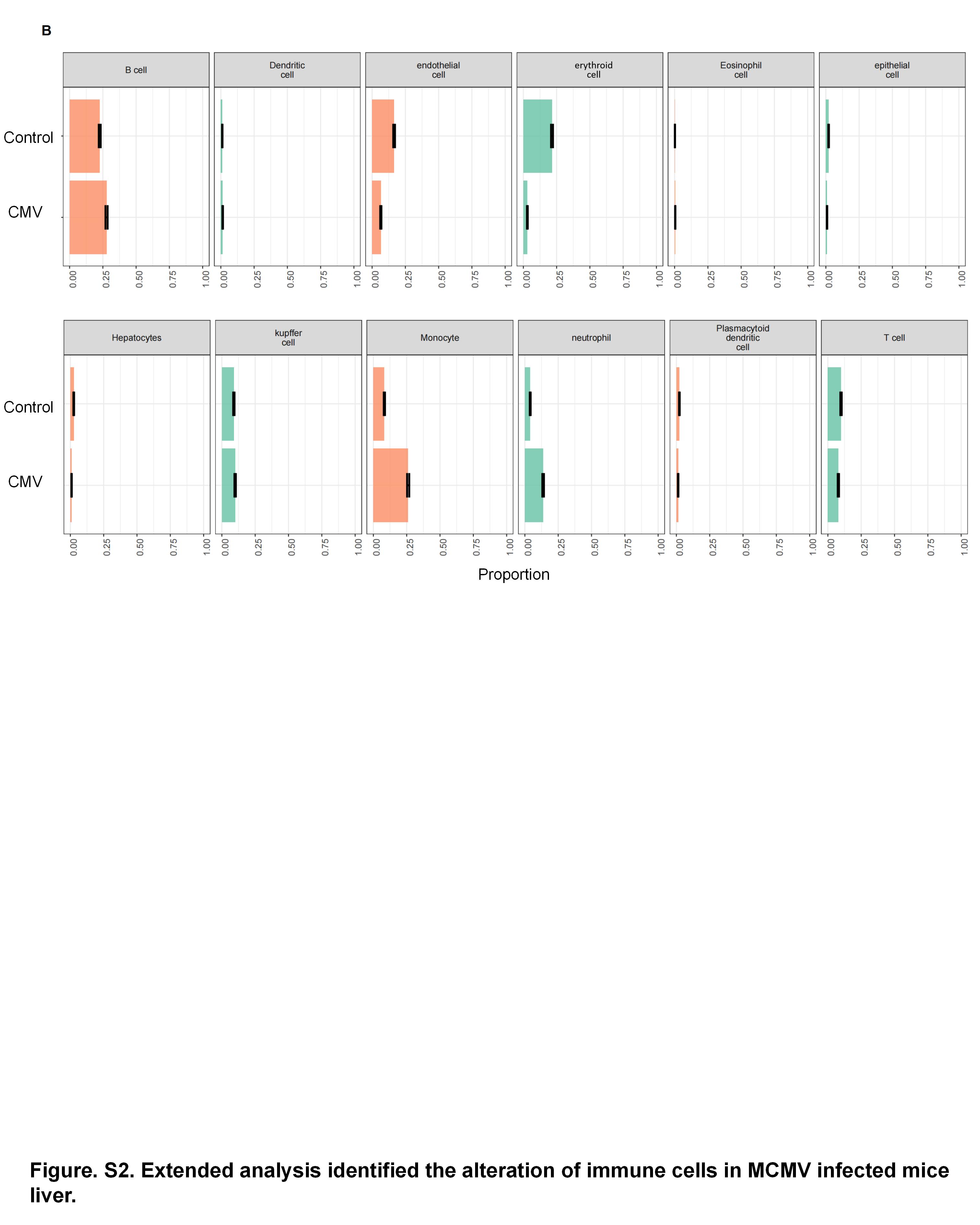

Supplement: Supplementary Figure 4 — Characteristic of Monocytes subclusters. (A) Expression of kupffer cell marker Clec4f of 4 clusters of myeloid cells in infant mice liver. (B) Distribution and expression of representative genes of 4 clusters of myeloid cells in infant mice liver. (C) The quantified percentage of each sub-cluster within Monocytes between uninfected and infected mice liver. (D) Distribution and exprssion of marker genes of all sub-clusters within Monocytes in infant mice liver. (E) Heat map of marker genes in 6 classical sub-clusters within Monocytes. (F) Distribution of Nos2 in main clusters of infant mice liver. (G-H) Pseudotime analysis of Monocyte cell sub-clusters in Monocyte cells from uninfected and infected infant mice liver by monocle2. (I) Representative gene expression heat map of two different cell fate in Monocyte analyzed by monocle2. (J) Cluster classical monocyte(Nos2 high) was selected to perform GO analysis between uninfected and infected mice. Enriched GO terms for significantly up-regulated genes were shown as bubble diagram. (K) Cell cycle scoring were conducted to view the proliferation status of each Monocyte sub-clusters. (L) The scDC analysis was conducted to statistically validate the alteration of cell percentage in scRNA-seq data. [file Image_4.jpeg]

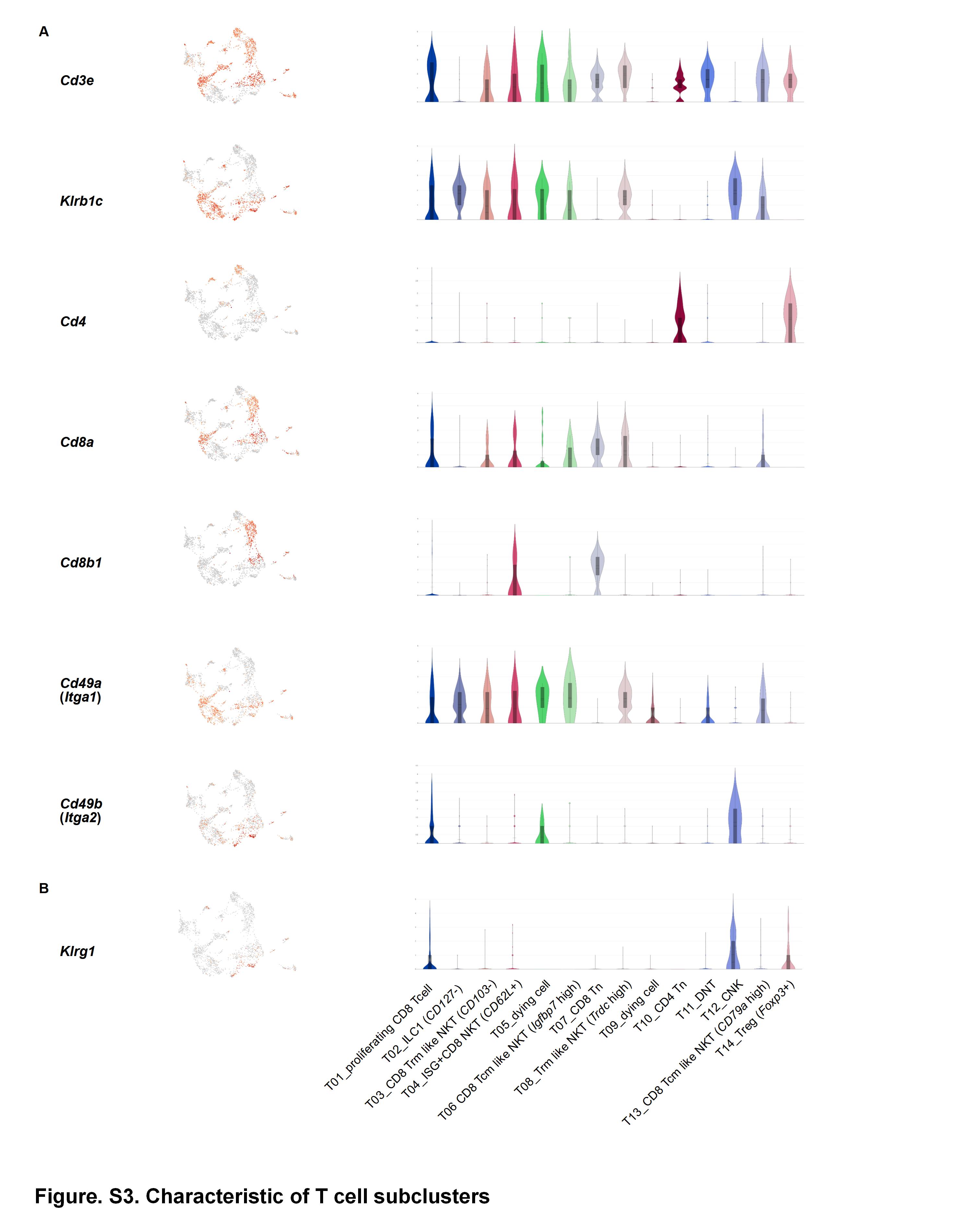

Supplement: Supplementary Figure 5 — Multi-omics analysis of MCMV infected mice liver. (A) Distribution and expression of representative enzymes of lipid metabolism in uninfected and infected mice liver, as shown by scRNA-seq data. (B) Enriched GO terms for significantly down-regulated genes of hepatocytes between uninfected and infected group in proteomic mass spectrometry analysis. (C) Enriched KEGG terms for significantly up-regulated genes of hepatocytes between uninfected and infected group in proteomic mass spectrometry analysis. (D) Enriched GO terms for significantly up-regulated genes of hepatocytes between uninfected and infected group in proteomic mass spectrometry analysis. [file Image_5.jpeg]

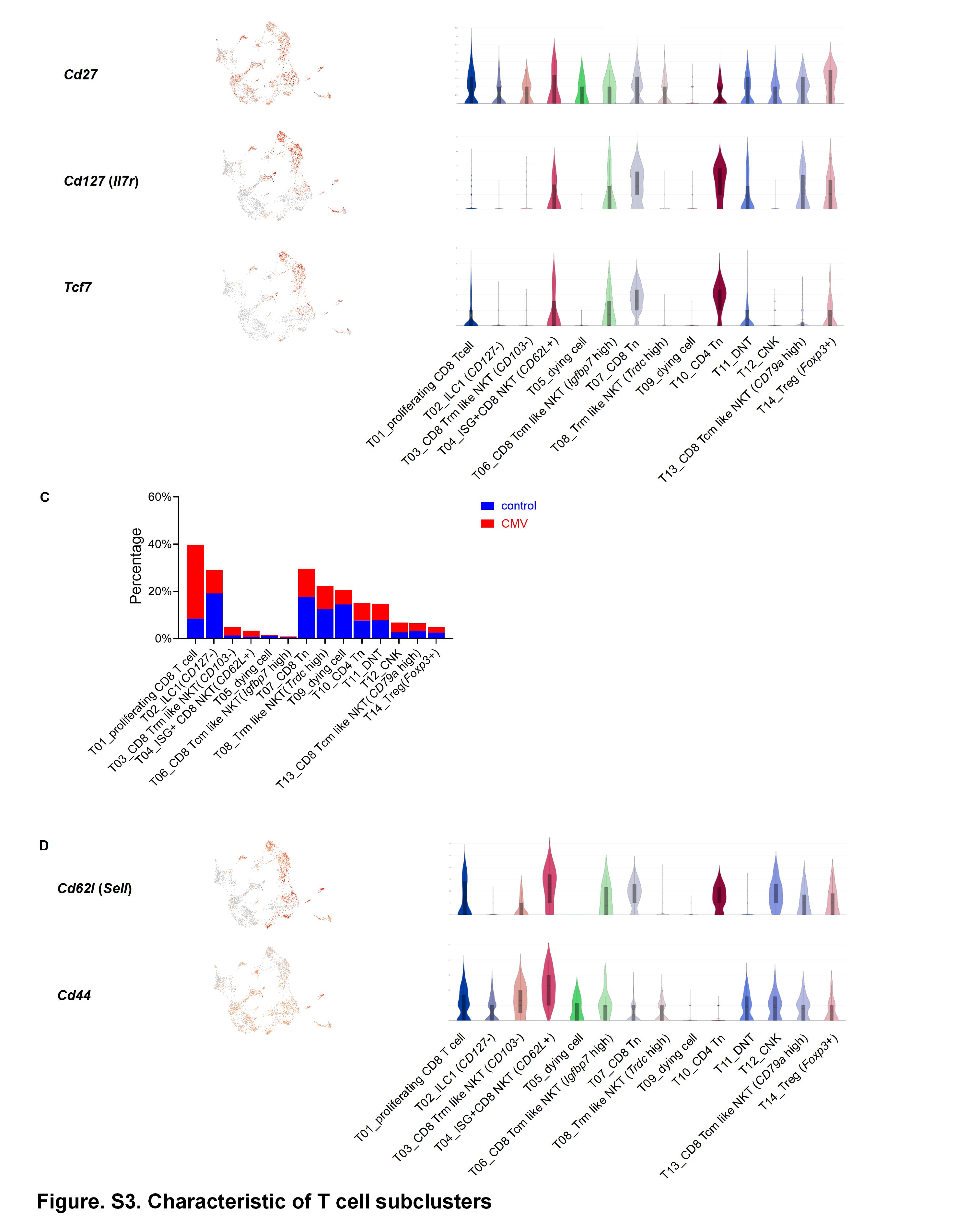

Supplement: Supplementary file 6 [file Image_6.jpeg]

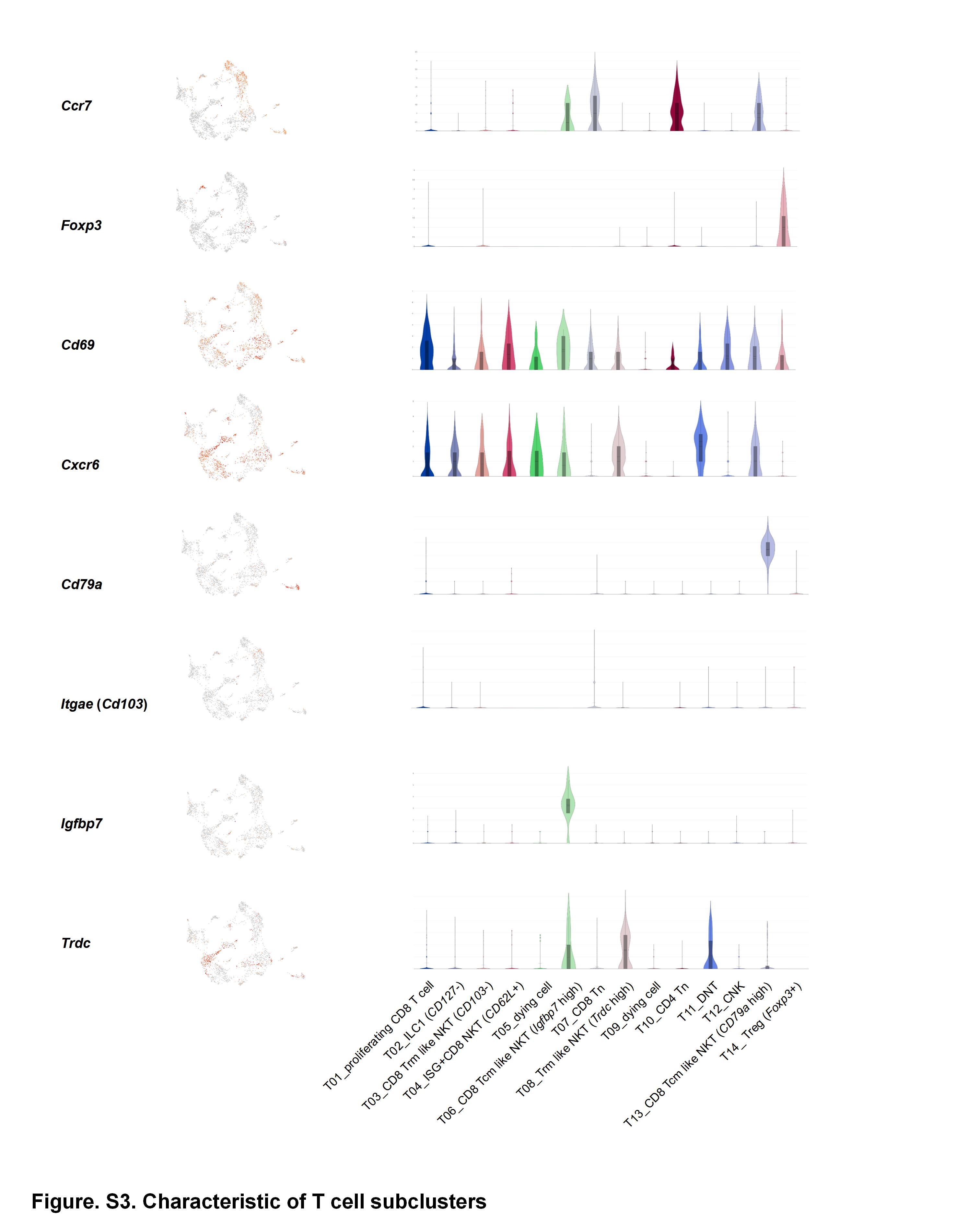

Supplement: Supplementary file 7 [file Image_7.jpeg]

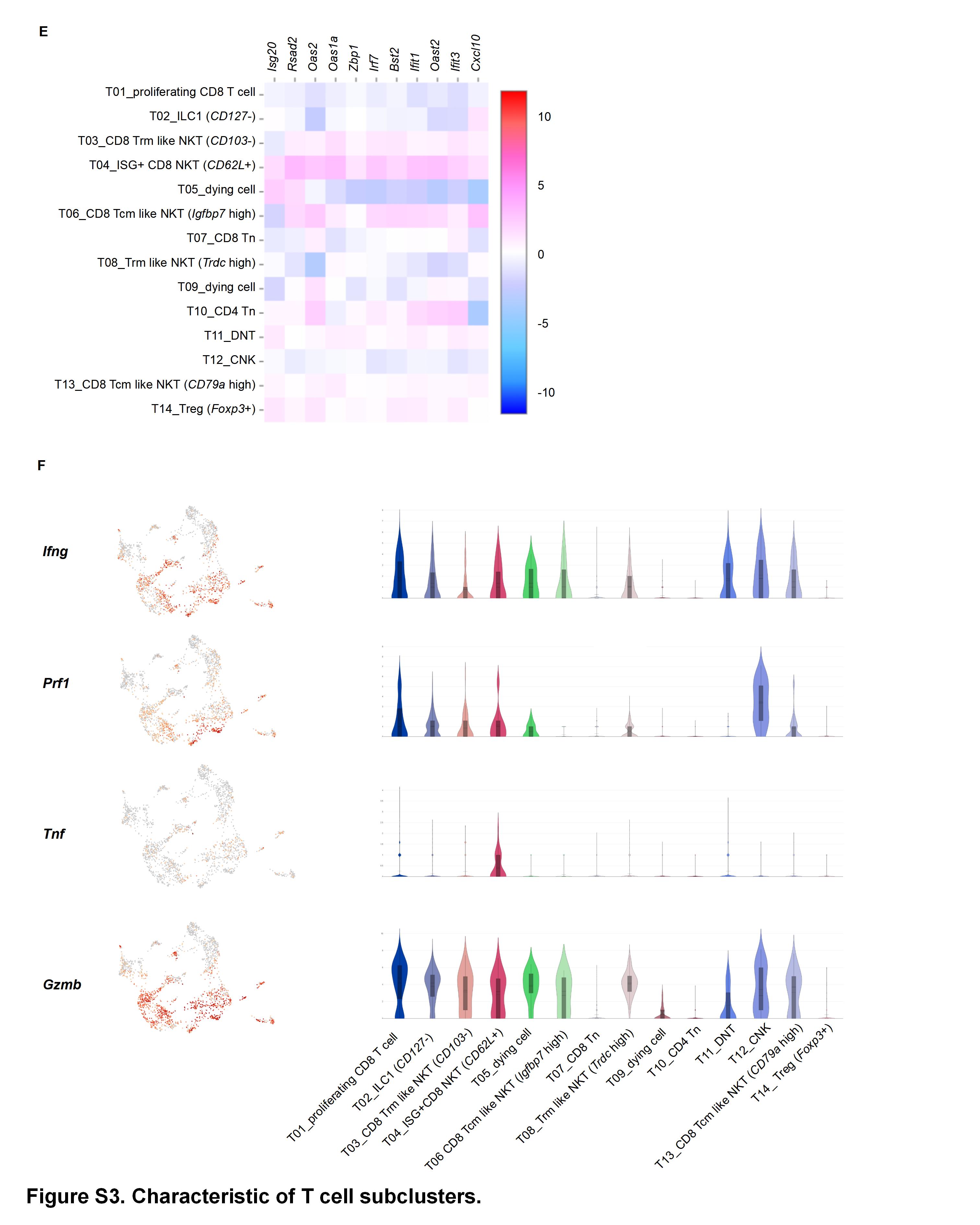

Supplement: Supplementary file 8 [file Image_8.jpeg]

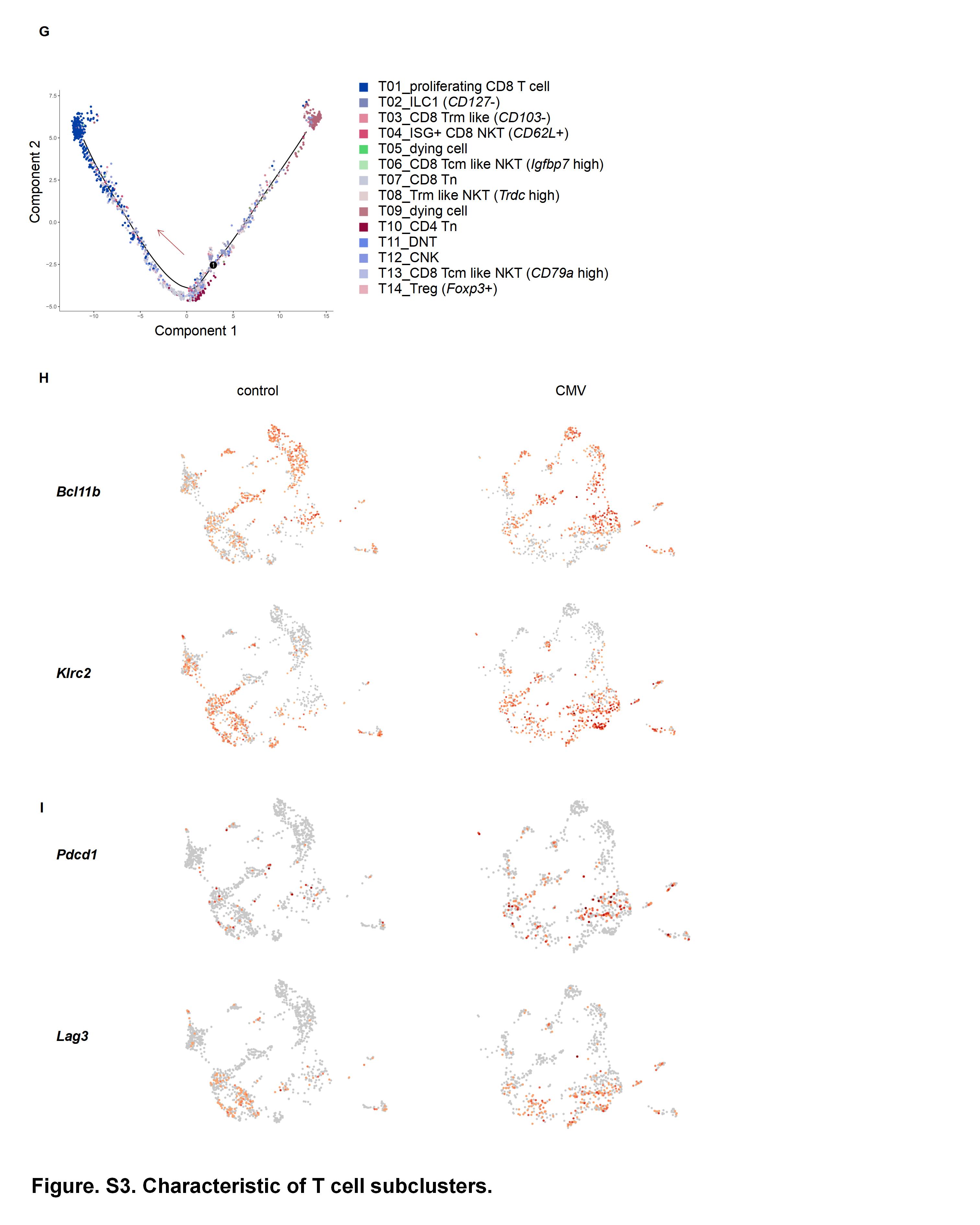

Supplement: Supplementary file 9 [file Image_9.jpeg]

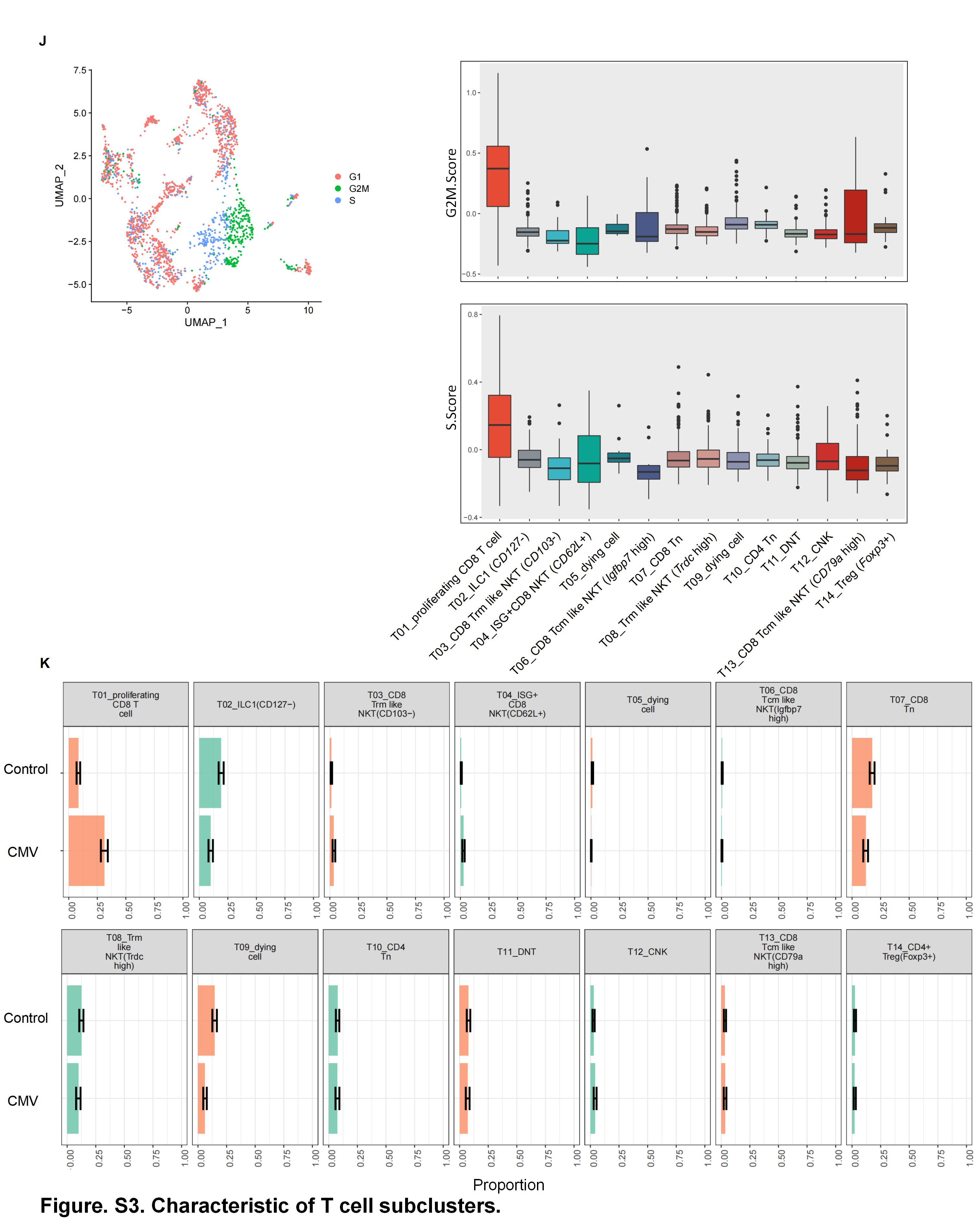

Supplement: Supplementary file 10 [file Image_10.jpeg]

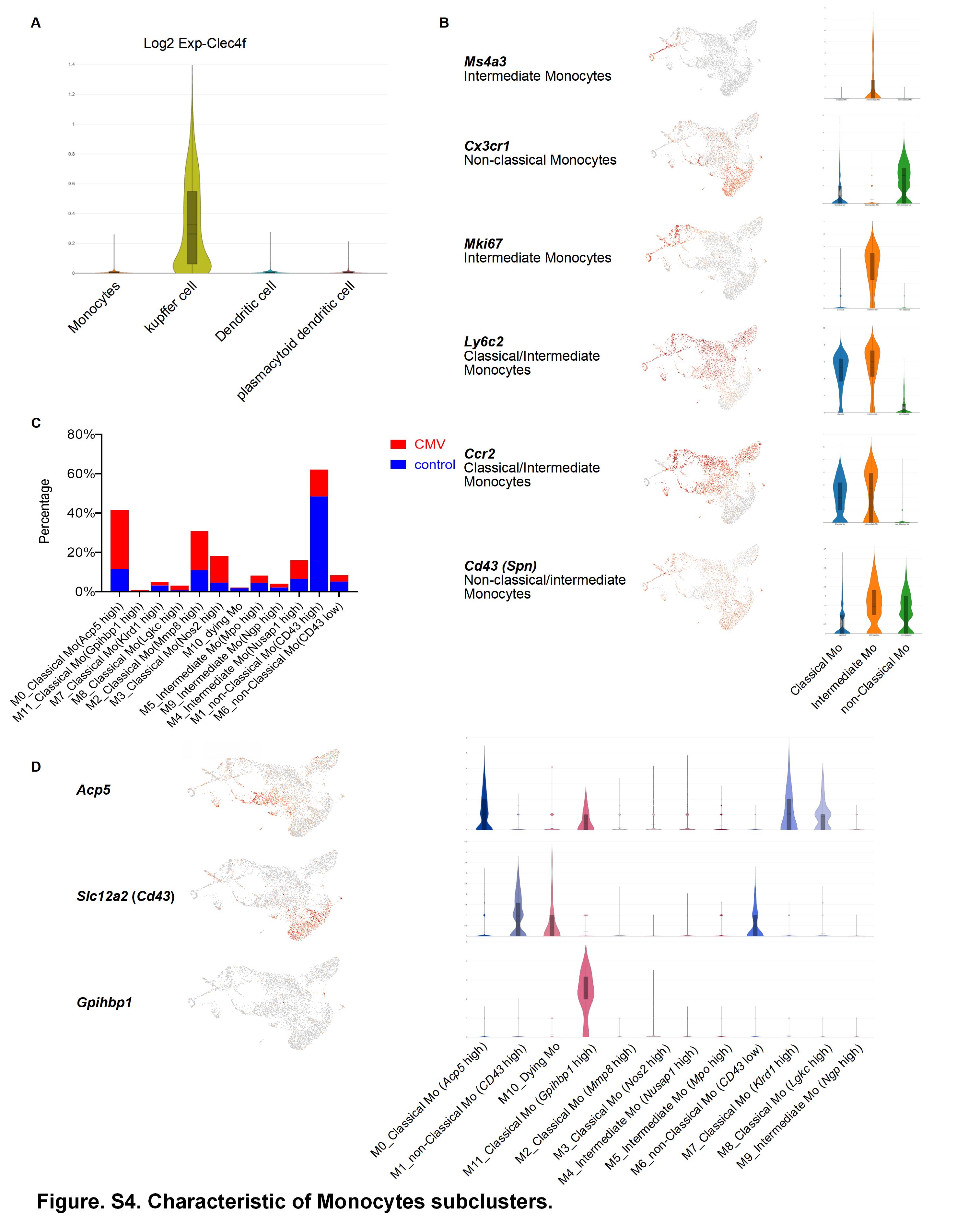

Supplement: Supplementary file 11 [file Image_11.jpeg]

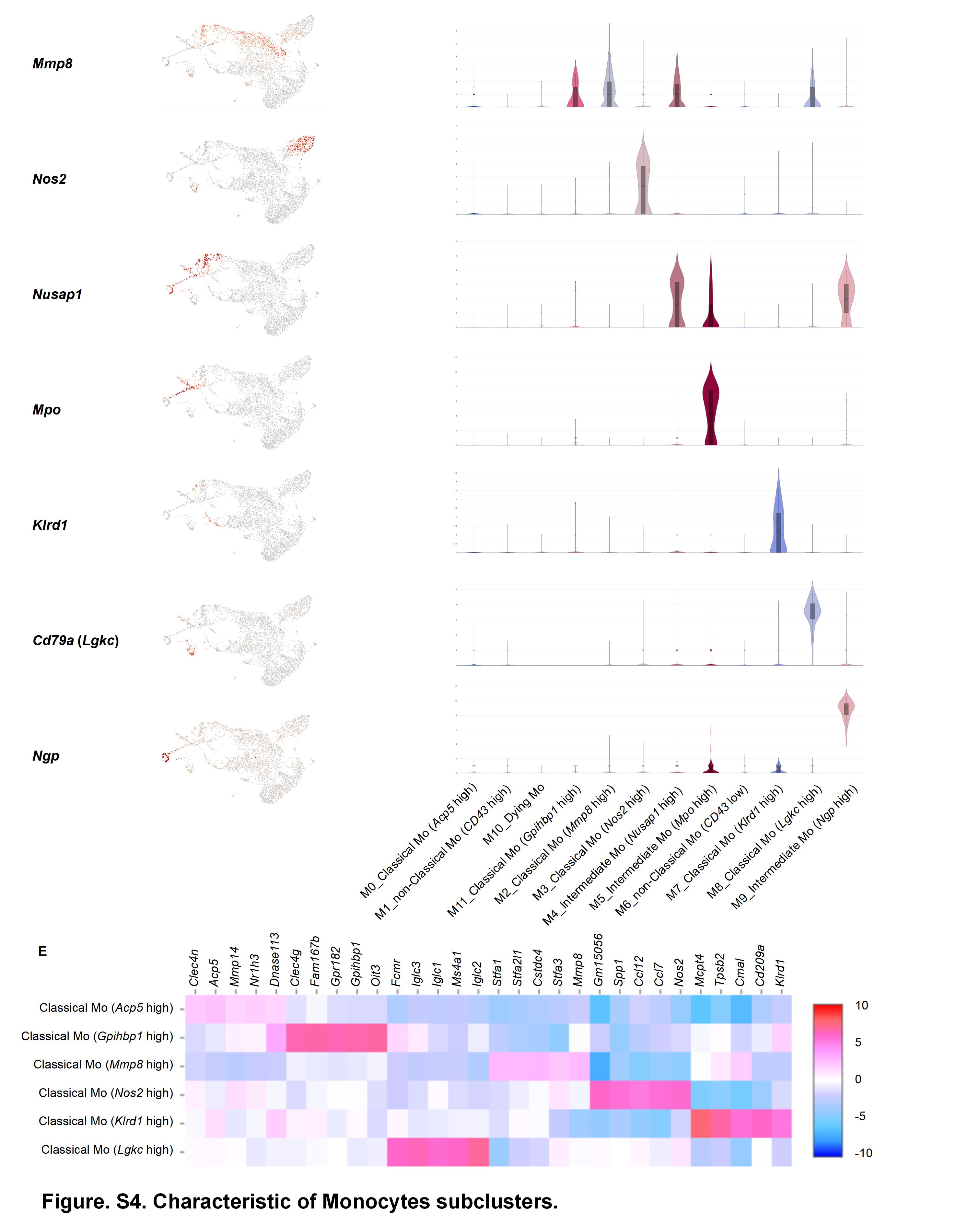

Supplement: Supplementary file 12 [file Image_12.jpeg]

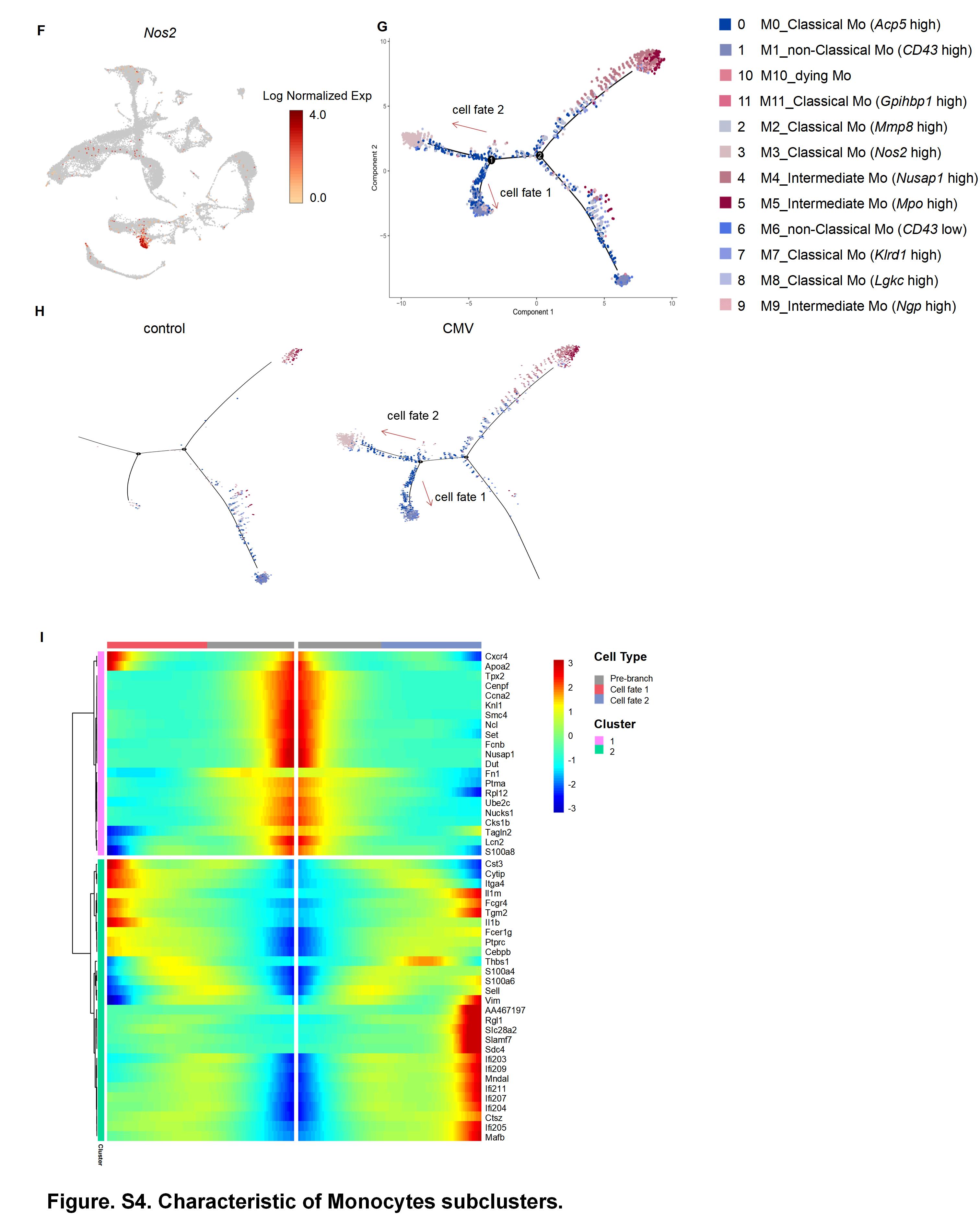

Supplement: Supplementary file 13 [file Image_13.jpeg]

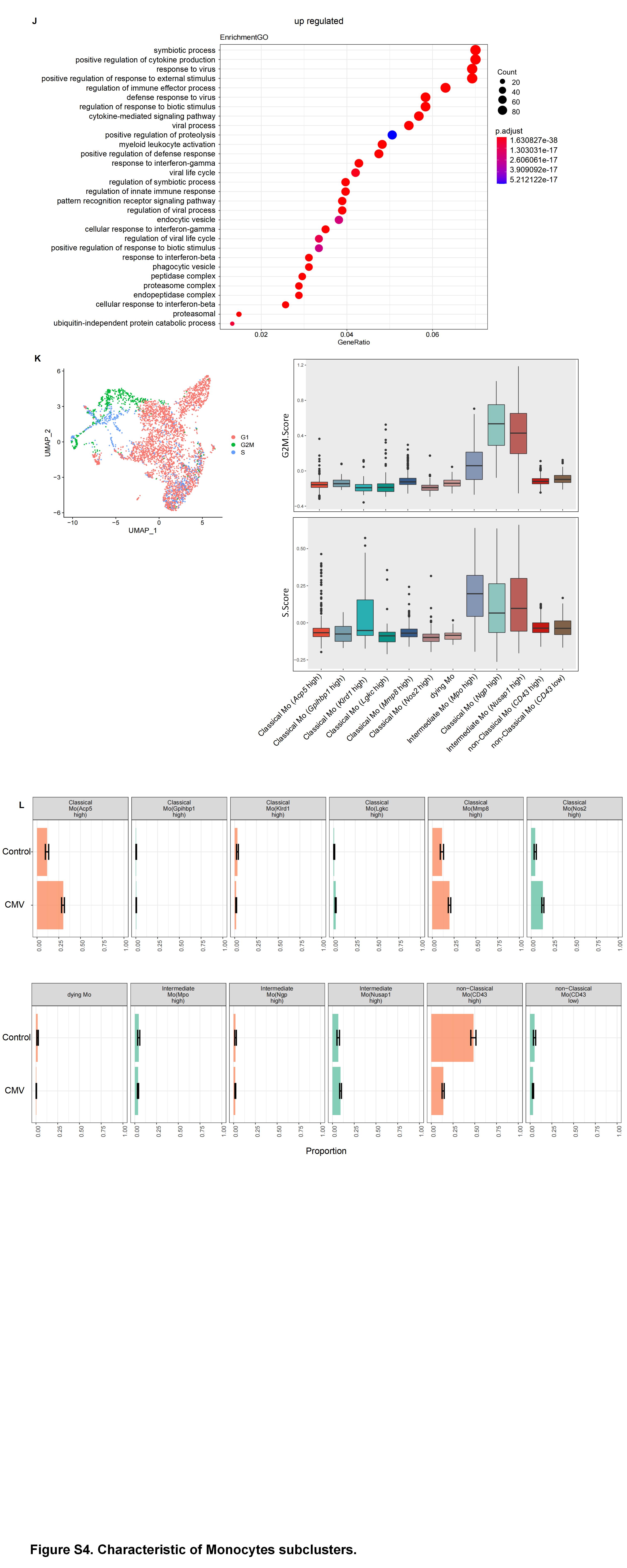

Supplement: Supplementary file 14 [file Image_14.jpeg]

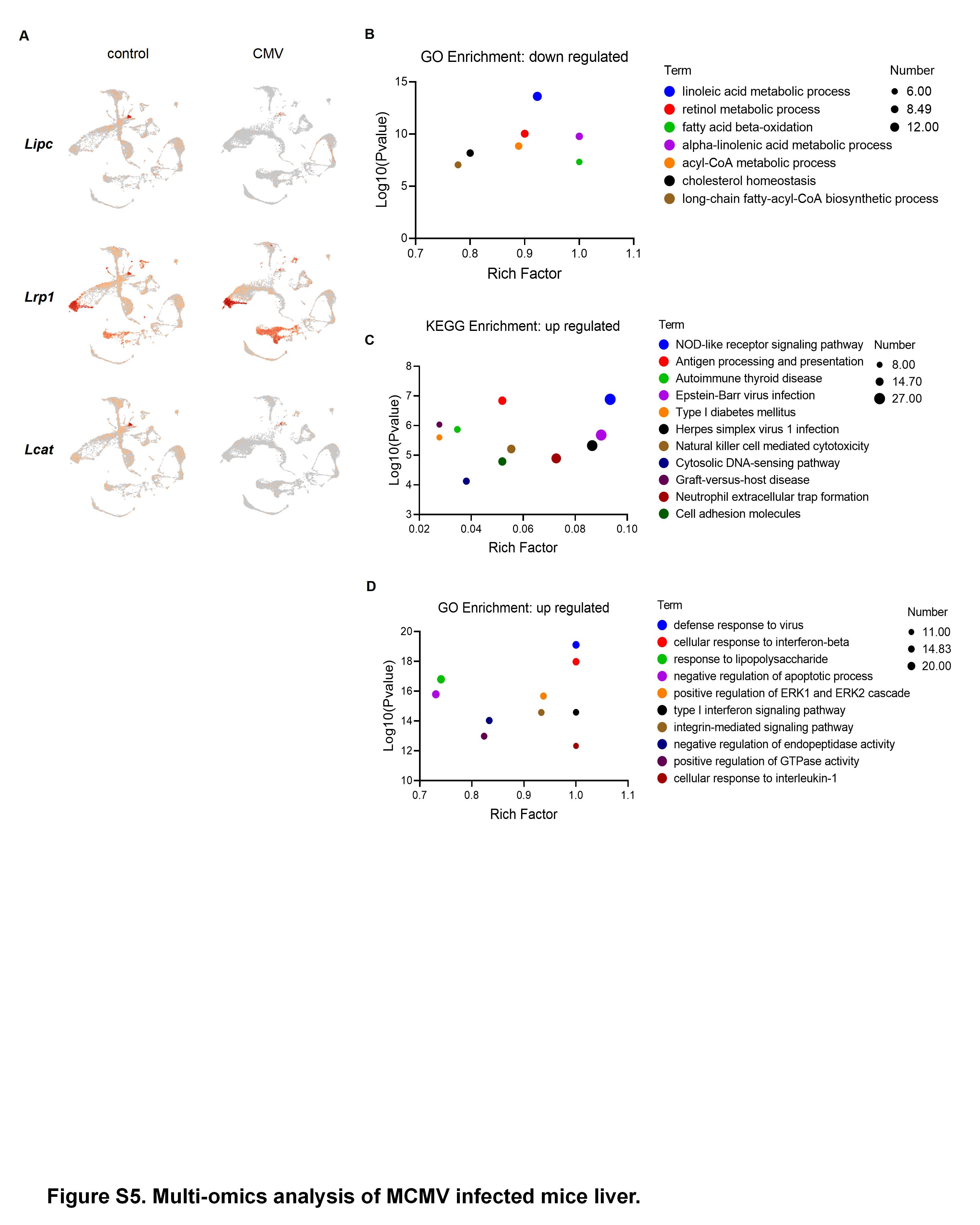

Supplement: Supplementary file 15 [file Image_15.jpeg]
